# On the Pore Geometry and Structure Rock Typing

**DOI:** 10.1021/acsomega.4c02879

**Published:** 2024-07-25

**Authors:** Farizal Hakiki, Muhammad Nur Ali Akbar, Zaki Muttaqin

**Affiliations:** † National Yang Ming Chiao Tung University, Disaster Prevention and Water Environment Research Center, Hsincu 30010, Taiwan; ‡ National Yang Ming Chiao Tung University, Civil Engineering Department, Hsincu 30010, Taiwan; § Griyanipun Tiang Sepah Kula, Malang 65153, Indonesia; ∥ Prores AS, Trondheim 7041, Norway; ⊥ An Independent Researcher, Rumah Saya, Tangerang 15810, Indonesia

## Abstract

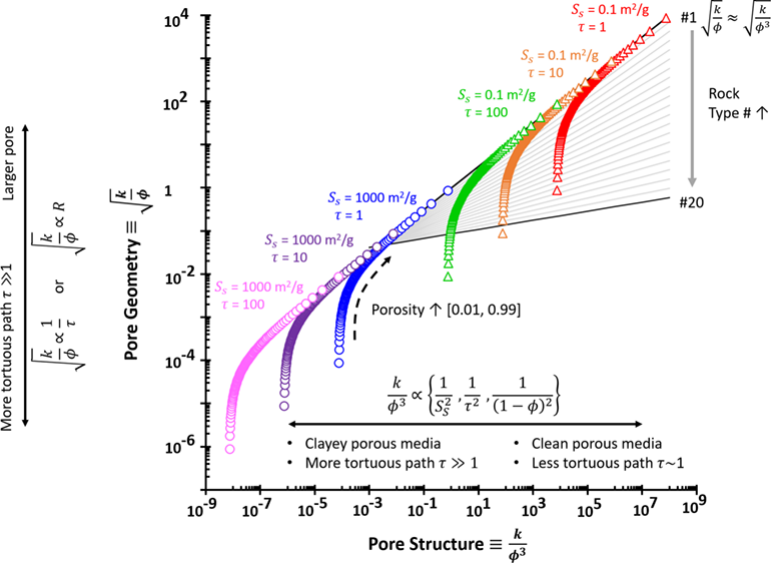

Rock typing is a vital step in oil and gas reservoir development to achieve predictions of hydrocarbon reserves, recovery, and underground storage capacity for CO_2_ or hydrogen. To address inaccurate initial hydrocarbon-in-place prediction and improper rock property distribution in a reservoir model, a recent rock typing method, pore geometry and structure (PGS), has revealed a more accurate prediction on connate water saturation and better grouping of capillary pressure. However, the current state still needs physical interpretations of the PGS rock typing. We have compiled thousands of experimentally measured hydraulic properties, such as permeability *k* within 12 orders of magnitude, porosity ϕ up to 0.9, specific surface area *S*_S_ within 4 orders of magnitude, and pore size *R* ranges around 3 orders of magnitude. We conduct the first-ever holistic physical interpretations of the PGS rock typing using gathered data combined with analytical theory and the Kozeny–Carman equation. Surprisingly, our physics-inspired data-driven study reveals advanced findings on the PGS rock typing. These include (i) why PGS method prevails over the hydraulic flow unit rock typing, (ii) explanations to distinguish between causality and indirect relationships among hydraulic properties, rock type number, and electrical resistivity, (iii) a proposed novel method: permeability prediction from the resistivity and rock type number relationship, and (iv) a suggestion and criticism on how to avoid a recursive prediction on permeability.

## 1 Introduction

Rock typing is a vital step in planning oil and gas reservoir development to achieve predictions on hydrocarbon reserves, recovery, and underground storage capacity for CO_2_ or hydrogen. Rock type distribution is critical for developing a static and dynamic geological model in a reservoir simulator. Parameters required in the model are, e.g., predicted permeability,^1,2^ water saturation,^3,4^ and the pay zone or cutoff definition.^5^

Classifying rocks based on their physical characteristics into a distinct unit is called rock typing. Samples in each class seem indistinguishable due to similar geological depositions and diagenetic alterations.^6^ Many methods have addressed rock typing, for example:

- Based on mechanical properties, mineralogy, and organic geochemistry^7^
- The use of permeability, porosity, and irreducible water saturation data either empirically^5,8−10^ or with hydraulic flow unit (HFU) approach;^1,3,11^
- Involvement of capillary pressure data and *J*-function^12−14^ and combined with radius;^5^
- Consideration of thin section descriptions and interpretations such as rock fabrics,^15^ depositional facies,^16^ and rock textures;^17,18^
- Geostatistics and machine learning implementation such as clustering,^19,20^ ANN,^21^ self-organizing map,^22,23^ and fuzzy logic;^24^
- Grouping based on the dimensionless form of absolute permeability, relative permeability, porosity, and phase viscosity, the so-called true effective mobility TEM function;^25,26^
- The use of resistivity data and porosity to yield in electrical flow unit;^27^
- Further development of analytical models, e.g., pore geometry and structure (PGS) method.^28−31^

PGS method is built on the basis of the Kozeny–Carman and capillary bundled model equations, ending up with a plot between  and *x* ≡ *k*/ϕ^3^ within a power function *y* = *ax*^*b*^(28,29) such that
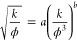
1

The inspiration came from the facts that many physical phenomena in nature are in a power law scaling relationship.^32^ Surprisingly, rocks under the same group of PGS rock type numbers (same constants *a* and *b*) reveal resemblant capillary pressure and *J*-functions^31,33^ because the *y*-component is actually used to deduce the pore geometry as described by the concept of Leverett’s *J*-function.^12,28−30^ Thus, PGS rock typing is an excellent tool to identify the pore size distribution: a larger pore size distribution PSD indicates a smaller rock type number (PSD ↑ → RT # ↓). Interestingly, there is also a good trend with the grain size distribution GSD measured by thin section: a larger grain size distribution GSD correlates with a smaller rock type number (GSD ↑ → RT # ↓).^34^ Note: The upward arrow ↑ indicates increased values. The downward arrow ↓ means decreased values. Meanwhile, the *x*-component designates the pore structure and accommodates tortuosity τ and specific surface area *S*_S_.^28^

The value of *b* is first determined from the lowest rock type number, named Rock Type #1, with the value of *b*_1_ = 0.4850 (the steepest power) and progresses as follows: *b*_*i*+1_ = *b*_*i*_ – 0.02 for the *i*th rock type. The selected value of Δ*b* = 0.02 refers to the statistical mode of the Δ*b* distribution, where each Δ*b* represents the slope changes in the log–log plot. Initially, the Δ*b* was statistically analyzed from 0 to 0.08 based on the existing carbonate samples.^31^ Later, we can compute the value of *a* with the following equation: *a* = 0.002^0.5–*b*^. The value of 0.002 indicates the origin point where all rock type lines converge into a coordinate of . Table 1 lists the exact values of *a* and *b*.

**Table 1:** Equation Parameters for the Pore Geometry and Structure PGS Rock Typing^a^

rock type # and boundary for rock type #	*a*	*b*
1	0.9110	0.4850
boundary	0.8673	0.4771
2	0.8045	0.4650
boundary	0.7660	0.4571
3	0.7105	0.4450
boundary	0.6764	0.4371
4	0.6274	0.4250
boundary	0.5974	0.4171
5	0.5541	0.4050
boundary	0.5276	0.3971
6	0.4893	0.3850
boundary	0.4659	0.3771
7	0.4322	0.3650
boundary	0.4115	0.3571
8	0.3816	0.3450
boundary	0.3634	0.3371
9	0.3370	0.3250
boundary	0.3209	0.3171
10	0.2976	0.3050
boundary	0.2834	0.2971
11	0.2629	0.2850
boundary	0.2503	0.2771
12	0.2321	0.2650
boundary	0.2210	0.2571
13	0.2050	0.2450
boundary	0.1952	0.2371
14	0.1810	0.2250
boundary	0.1724	0.2171
15	0.1599	0.2050
boundary	0.1522	0.1971
16	0.1412	0.1850
boundary	0.1344	0.1771
17	0.1247	0.1650
boundary	0.1187	0.1571
18	0.1101	0.1450
boundary	0.1048	0.1371
19	0.0972	0.1250
boundary	0.0926	0.1171
20	0.0859	0.1050
boundary	0.0818	0.0971
21	0.0758	0.0850
boundary	0.0722	0.0771
22	0.0670	0.0650

a (1) Initial power of rock type *b* at the steepest slope = 0.4850. (2) Differences between two consecutive rock types # Δ*b* = 0.02. (3) Initial power of the boundary *b*_*b*_ at the steepest slope = 0.4771. (4) Differences between two consecutive rock type # boundaries Δ*b*_*b*_ = 0.02. (5) Both rock type and boundary line initial values are computed as follows: *a* = 0.002^(0.5–*b*)^, *a*_*b*_ = 0.002^(0.5–*b*_*b*_)^.

To date, no comprehensive experimental results confirm the distribution of pore radius *R*, tortuosity τ, and specific surface area *S*_S_ on PGS rock typing despite the broad use of the method for sandstones^35^ and carbonates.^35−37^

In addition, there are no explanations yet that answer: (a) Why does PGS method work better than HFU approach in grouping capillary pressures;^38^ (b) Why does the predicted permeability with PGS method always fit well compared to explicit equations, such as Tixier,^39^ Coates,^40^ and Timur^41^ (examples found in refs (42−46)).

Herein, we holistically investigate the distribution of pore radius *R*, tortuosity τ, and specific surface area *S*_S_ on PGS method compared with the Kozeny–Carman equation and measured data from unconsolidated geomaterials (coarse to fine grain soils), sandstones, carbonates, and tight rocks. The wholistic data address unanswered questions and extend further applications related to PGS method. We also provide detailed derivations of involved equations in this research (Supporting Information: Appendices A–E).

## 2 Methods

We compile thousands of data points for soils (sandy, silty, and clayey soils) and rocks (sandstones and carbonates). The data compilations encompass laboratory-measured and field properties from well-logging, i.e., resistivity.

Most soil data are in different units compared with rocks; thereby, conversion is required. The conversion formula from geotechnical engineering to petroleum engineering field comprises hydraulic conductivity *k*_h_ [m/s] to permeability *k* [mD] and void ratio *e* [] to porosity ϕ []:
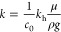
2
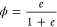
3with specified values as follows: water viscosity μ of 0.001 kg/m/s, water density ρ of 1000 kg/m^3^, gravity *g* of 9.8 m/s^2^, and unit converter *c*_0_ = 9.869233 × 10^–16^ m^2^/mD. Appendix A describes the derivation of equations related to fluid flow in porous media from the first principle of Newton’s viscosity law and equates the results with Darcy’s equation.

Other compiled data are the pore diameter *d*, radius *R*, and gravimetric specific surface area or shortly mentioned as specific surface *S*_S_. Some published works prefer a volumetric-bulk specific surface area *S*_V_ or volumetric grain-specific surface area *S*_Vg_; therefore, we need to perform conversions (details in Appendix B). We carefully check the data we gather to see if they adhere to the physics-guided equations (Appendix A), such as
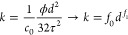
4where *f*_1_ ≈ 2 (eq A-9b in Appendix A), and the Kozeny–Carman equation:
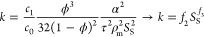
5where *f*_3_ ≈ −2 (eq C-3 in Appendix C), variables and units in eqs 4 and 5 are *d* [m], *k* [mD], ϕ [], τ [], ρ_m_ [g/cm^3^], *S*_S_ [m^2^/g], and the unit converters are as follow: *c*_1_ = 10^–12^ m^6^/cm^6^ and *c*_0_ = 9.869233 × 10^–16^ m^2^/mD. Geometric factor α that determines the pore cross-sectional shape exhibits negligible effects; varying values: 2-4. See eqs B-7, B-8, and B-9: α = 2 for parallel sheet pores (e.g., kaolinite) and α = 4 for cylindrical pores.

Specific surfaces of in-situ soils are fabric- and stress-dependent. Therefore, we assume that specific surfaces measured under atmospheric conditions are adequate (the methylene blue method in ref (47), image analysis method in ref (48)). Standard methods for specific surface determination in rocks include BET methods with N_2_, Ar, or He gases or from analysis of mercury intrusion data.^49,50^

Unlike rocks that are measured using mercury intrusion, the pore size measurements in clayey soils are physically impossible due to swelling phenomena. Thence, most soils’ pore sizes *d* are determined with the deduction from the specific surface area,  or with a geometric factor α inclusion (see Appendix B).

**Figure 1:**
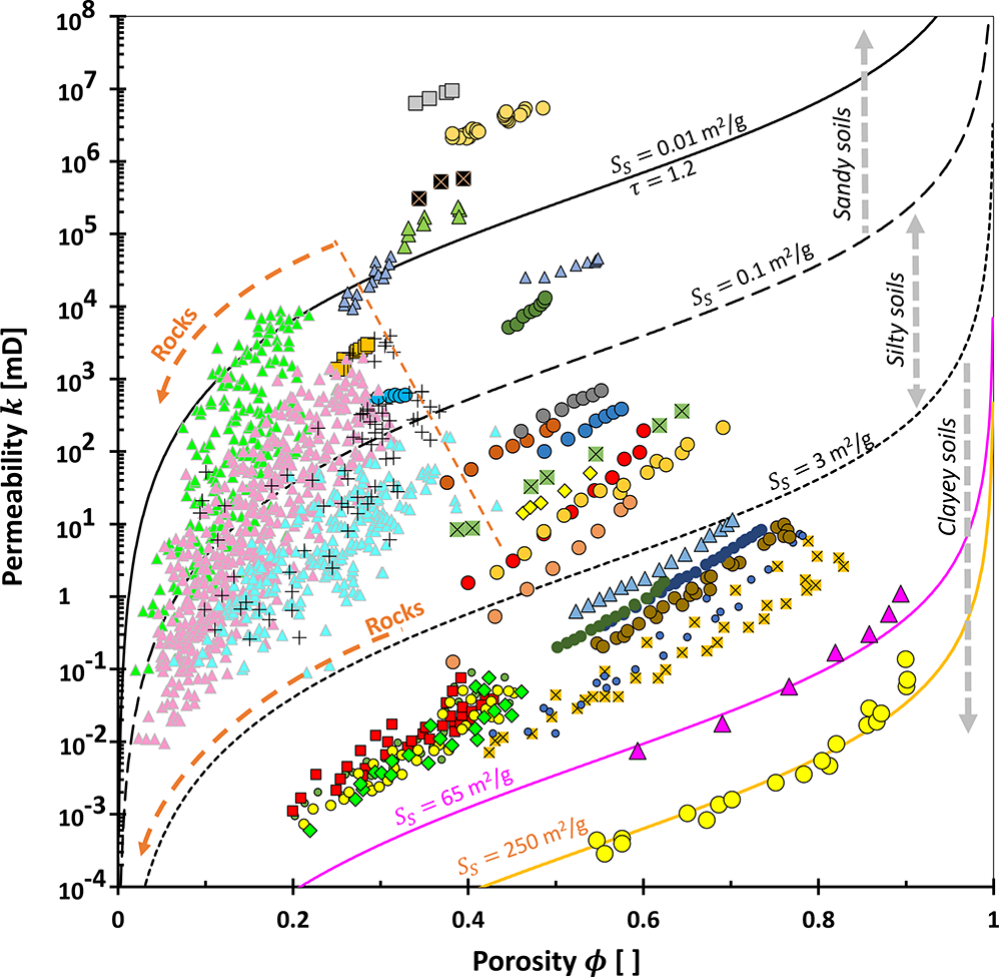
Hydraulic properties map of porous media: permeability vs. porosity. Color codes denote distinct samples. Descriptions and data sources**:** Soils include coarse to fine sediments with varied fabrics.^51^ The conversion of soil hydraulic conductivity (m/s) to permeability (mD) assumes water-filled soil with the following properties: viscosity of 0.001 kg m^–1^ s^–1^, density of 1000 kg/m^3^, and gravity of 9.8 m/s^2^. Sandstone data appear as black +.^28,52^ Carbonates according to Lucia’s classification; data: refs (53,54) in ref (50) grainstones (pastel green triangle), packestones (pastel pink triangle), and wackestones-mudstones (pastel blue triangle). Fitting lines use the Kozeny–Carman equation with the following assigned values: hydraulic tortuosity τ of 1.6 (unless stated), mineral density of 2.6 g/cm^3^, and specific surface area *S*_S_ adjacent to the lines.

Figure 1 curates selected data compilations from soils, sandstone, and carbonate rocks using the permeability-porosity map and Kozeny–Carman fittings. We highlight a typical window of rocks that spans porosity ϕ ≤ 0.4 and permeability *k* between 10^–2^ and 10^4^ mD (orange dashed lines). Rocks may lie within the sandy and silty soil ranges. However, sandy and silty soils themselves exhibit higher porosity ϕ > 0.3. Clearly, the specific surface area *S*_S_ plays a critical role in determining the window of sandy (*S*_S_ ≤ 0.1 m^2^/g), silty (0.1 m^2^/g < *S*_S_ < 3 m^2^/g), and clayey soils (*S*_S_ ≥ 3 m^2^/g).

## 3 Results and Discussion

### 3.1 Theoretical Frameworks

**Figure 2:**
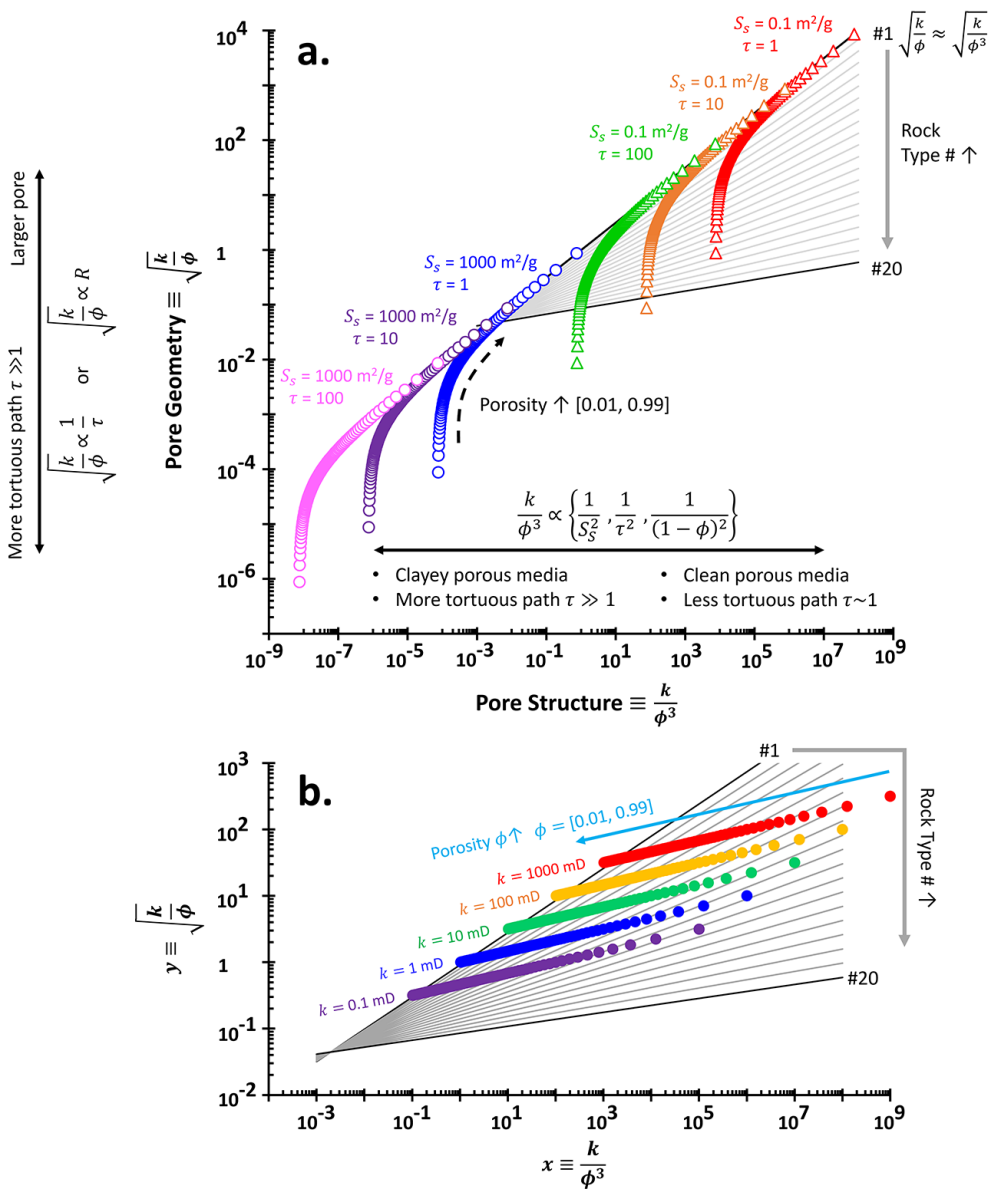
Theoretical framework of the rock typing: Pore geometry and structure. (a) Physical interpretations of the permeability computed using the Kozeny–Carman equation. Each color code spans the porosity range of 0.01–0.99 and with a given specific surface area *S*_S_, hydraulic tortuosity τ, geometric factor α = 4 (cylindrical pores), and constant mineral density ρ_m_ of 2.6 g/cm^3^. Written rock typing numbers: RT #1–20. (b) Distributions of porosity-permeability on rock typing in the case of either constant porosity or permeability only and independent.

We revisited the Kozeny–Carman equation on the PGS style plot, i.e., between  and *x* ≡ *k*/ϕ^3^ (Figure 2a). This study aims to theoretically expose possible limitations of PGS method. The data in Figure 2 may not physically exist and are used to explore what mathematical expressions can do. Let us define the “pore geometry”  which is equivalent to the rearranged eq 4 and derived from the combinations of Newton’s viscous law, capillary bundled model, and Darcy’s equation:
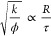
6

It is proportional to the radius *R* and inverse of tortuosity 1/τ. The *y*-component of Figure 2a justifies that the more tortuous rocks 1/τ ≪ 1, the higher the rock type number (RT # ↑). Conversely, the larger the pore radius *R*, the smaller the rock type number (RT # ↓). This *y*-axis analysis elaborates on the implicit roles of how fluids flow easily in smaller rock types. One core sample typically suggests a single defined tortuosity and multiple values of pore size, which was later named the pore size distribution.

Let us now consider the permeability *k* over cubic porosity ϕ^3^ from the Kozeny–Carman equation, which we define as the “pore structure” in Figure 2:
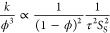
7

This implies that permeability is implicitly dominated by tortuosity τ and gravimetric specific surface area *S*_S_. We exclude the porosity ϕ discussion due to its involvement as an explicit variable (on the left-hand side). If we look at a constant *y*-value in Figure 2a, the smaller *x*-component shows that rocks belong to the smaller rock type number RT # ↓ due to both clayey *S*_S_ ↑ and tortuous τ ≫ 1 material.

Consequently, the rock type number indicates a contradiction in the tortuosity point of view between *x*- (τ ↑ → RT # ↓) and *y*-components (τ ↑ → RT # ↑). Notwithstanding the conflicting notions, the rate of tortuosity influence in the *x*-component is twice that of the *y*-axis due to the exponent, *k*/ϕ^3^ ∝ 1/τ^2^. Simply, we can say that geomaterials with more tortuous paths τ ≫ 1 would appear toward the southwest direction of the plot. Less clayey materials and larger pores would lay more onto the northeast trend of the chart.

Based on the Kozeny–Carman equation analysis, there is no evidence that each rock type number corresponds to a group of rocks that exhibit a similar tortuosity τ, unless the porosity ϕ is more than 0.7 (Figure 2a). This statement is made based on purely mathematical analysis in which the method to define tortuosity τ may still be impugnable. Further analyses based on selected typical values of tortuosity {1.3, 2, 4} and specific surface area {0.001, 0.1, 1, 10, 100} m^2^/g also make it difficult to conclude that a nearby rock type number designates a similar tortuosity (Appendix F). Physically, rock cores with different rock type numbers may have their own corresponding tortuosity. However, the range of the typical tortuosity is so narrow that it is difficult to have a distinct tortuosity. Note: Appendix D proves that tortuosity is approachable from the electrical resistivity.

Interestingly, geomaterials would fall approximately onto  for ϕ ≥ 0.7. Examples of very porous geomaterials are rocks with high diatomite content^49^ or loose soils.^51^ This proves that the highest physically admissible values of *a* ≈ 1 and *b* = 0.5 regardless of the tortuosity, pore shape (expressed by the geometric factor α; eqs B-9 and C-4), and specific surface area. In the extreme case, let *a* = 1 and ϕ = 1, the mathematical operation on eq 1 provides *b* = 0.5. Consequently, we should not interpret “The values of *a* = 1 and *b* = 0.5 correspond to the straight capillary tubes” as described in the earlier literature.^31^ The latter reference should claim the other way around: “Porous medium with straight capillary tubes is a subset of the case where *a* = 1 and *b* = 0.5”.

Independent permeability-porosity analysis in Figure 2b demonstrates that a higher porosity results in a smaller rock type number. These data are generated without considering the Kozeny–Carman equation because both properties are explicit variables. If we select a pivot value in the *x*-axis, we can see that a smaller rock type number follows a higher permeability. To that end, higher permeability and porosity corroborate that they are in a smaller rock type number. Interestingly, samples with porosity ϕ ≥ 0.7 tend to be around  regardless of the permeability.

It sounds impossible for a specimen to have varied permeability values smaller than 1 mD for a high porosity value. Nevertheless, results in Figure 2b act as the sanity check and limit the highest physically admissible power value for the PGS rock typing, i.e., *b* = 0.5.

### 3.2 Experimental Evidence

Our compiled data and Kozeny–Carman fittings in Figure 1 are contrary to a previous statement:^55^ “Kozeny–Carman equation is suitable for sandy soils to model hydraulic conductivity, but it cannot be applicable for clayey soils”. Our data support those of later studies that use Kozeny–Carman for clayey soils^56^ and reveal that the Kozeny–Carman equation is a valid model for various geomaterials, from low to high clay content and from rocks to soils.

We investigate two properties related to PGS rock typing: pore size and specific surface distribution. We do not consider tortuosity because this attribute is not measurable. There are a few methods to quantify tortuosity, such as based on electrical-tortuosity derived from resistivity,^57^*J*-function,^14^ permeability, porosity, specific surface area, and pore size distribution relationships,^58,59^ and micro-CT analyses.^60^ However, the validation of tortuosity values remains unclear. It is delicate to define the fluid path length, as pores could branch out and merge due to the presence of many possible paths inside the pores from the inlet to outlet.

**Figure 3:**
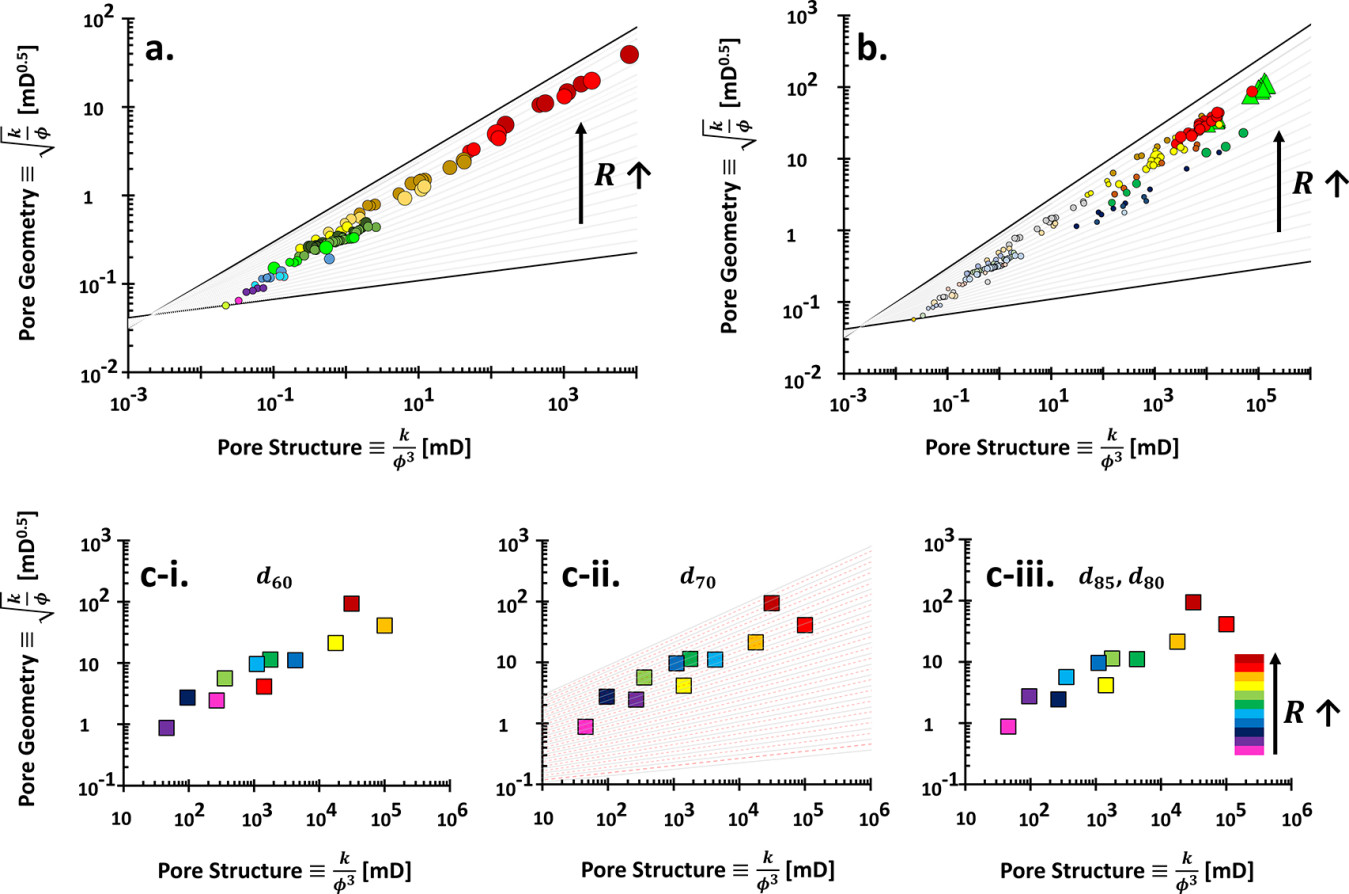
Pore geometry and structure rock typing with varying pore sizes. (a) Soils, (b) Sandstone, and (c) Carbonate rocks. Colors in (a,b) denote the rock typing number. However, the colors in (c) show the size of the pore diameter and are also marked by the legend in (c-iii). The bullet size in (a,b) illustrates the pore size. Descriptions and data sources: Soil data.^61^ Pore space *d*_p_ of soil is computed with the following eq B-7: , where specific surface area *S*_S_ is measured by methylene blue or gas adsorption. Sandstone data are portrayed by the distribution of the apex diameter *d*_apex_ (triangles^52^) and the mean distribution *d*_mean_ (circles^28^). Carbonate data are obtained by varying selected percentile diameters from *d*_60_ to *d*_85_.^50^

The pore size distributions mapped in Figure 3a,b highlight that the larger pore sizes result in a smaller rock type number (RT# ↓) and are also consistent with the theoretical results in Figure 2. These data pervade soils (sandy, silty, and clayey soils) and rocks (sandstones and carbonates). Carbonate rocks data in Figure 3c does not show a varied rock type number due to a limited number of data, and most are between RT #4 and #8—however, the larger the pores, the higher the “pore geometry” values. Figure 3c-iii also exposes a special notice for carbonates: d80 and d85 are the most representative pore sizes, which adhere to the theory developed in Figure 2.

**Figure 4:**
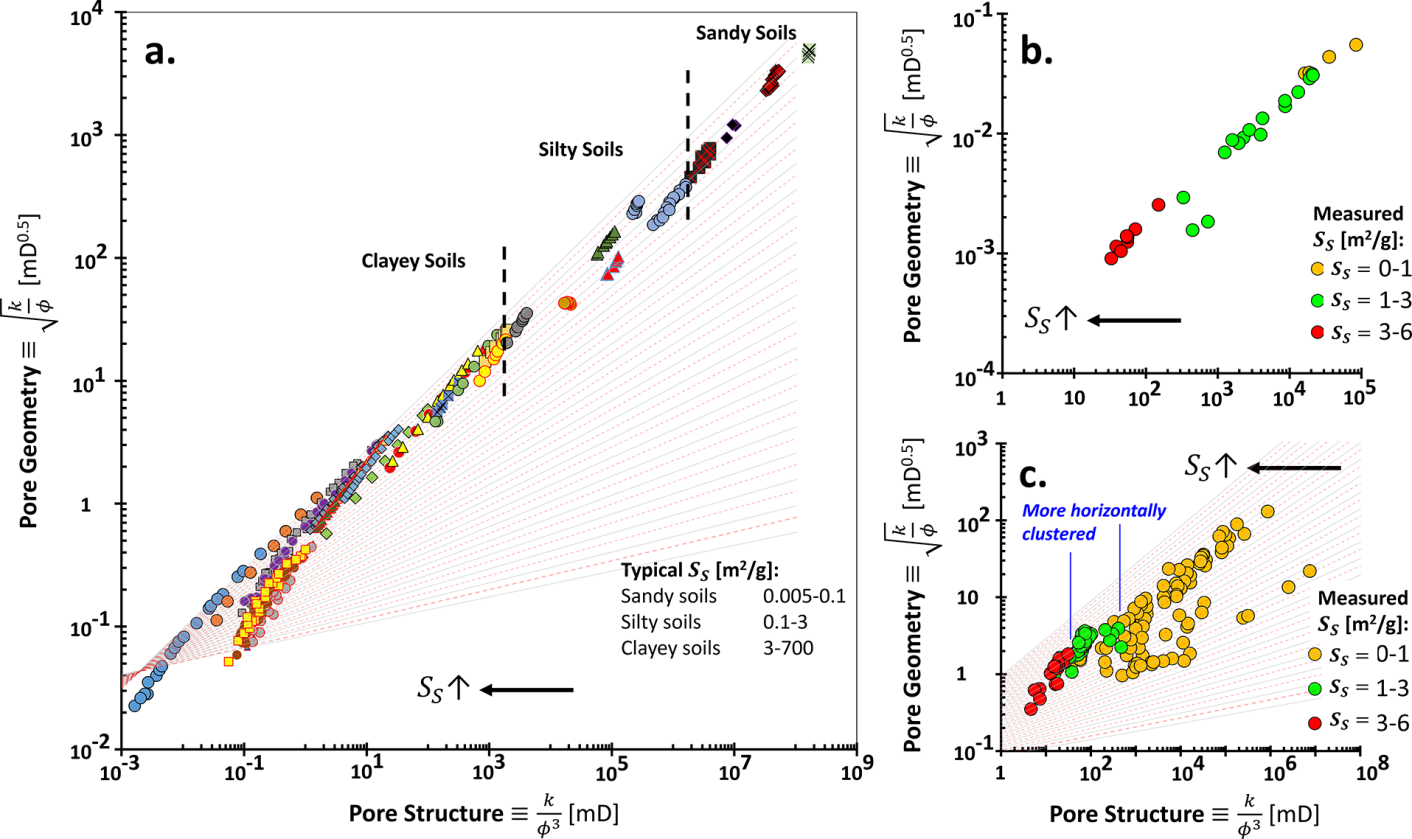
Pore geometry and structure rock typing with varying specific surface areas. (a) Soils, (b) Sandstone, and (c) Carbonate rocks. Descriptions and data sources: Specific surface area *S*_S_ of soils is measured by methylene blue or gas adsorption. Soils are abstracted from 23 earlier references and include sandy, silty, and clayey types.^51^ The specific surface area of sandstone is measured by nitrogen and argon adsorption.^62^ Detailed carbonate rocks include limestone,^62^ dolomites, limestone, and chalks,^63^ chalks,^64^ chalks,^65^ and neritic carbonate.^66^

The distribution of gravimetric specific surface *S*_S_ in Figure 4 is also supportive of the theoretical work in Figure 2. The geomaterials with higher specific surfaces lay onto more southwest distribution on the plot due to lower “pore structure” values. Coarser soils tend to be in between Rock Type #1 and #5, e.g., silty and sandy soils. Meanwhile, the finer soils, such as clays reach rock type class higher than RT #5 with much lower “pore structure” values <10.

**Figure 5:**
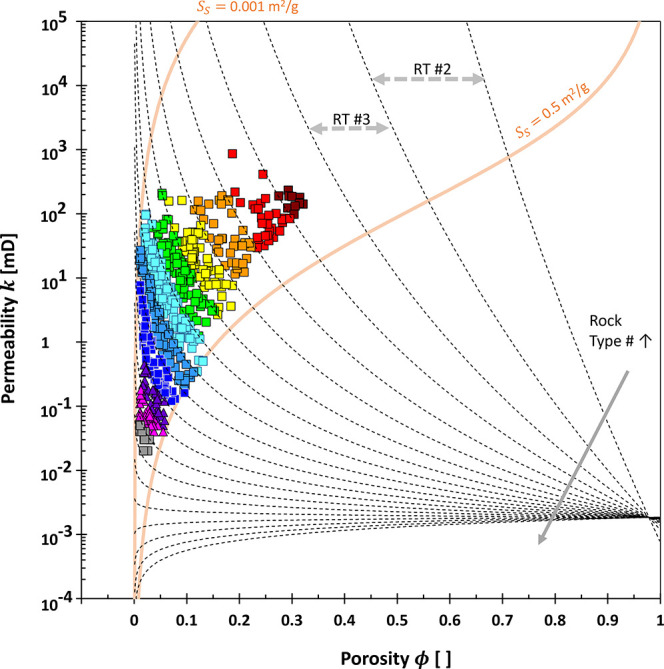
Pore geometry and structure PGS rock typing in the permeability-porosity map. Color codes represent each rock type number RT # (rock type unit based on PGS method). Data are from carbonate rocks.^24^ The light-brown lines are the Kozeny–Carman equations with the following properties: declared specific surface area *S*_S_, hydraulic tortuosity τ of 1.3, and a rock density of 2.6 g/cm^3^. The dotted lines refer to the rock type boundary lines.

Figure 5 visualizes the PGS-clustered carbonates onto a conventional permeability–porosity plot. Each rock type number (each color code) indicates that the higher porosity, the permeability becomes reduced. The Kozeny–Carman equation answers this relationship; i.e., the higher porosity, the rock distribution in each rock type is evolving into a higher specific surface area *S*_S_ (in the southeast direction). This higher specific surface area is theoretically proportional to a smaller pore size ( in Appendix B). Therefore, the PGS plot assists in the identification of the representative pore size and specific surface area.

### 3.3 Rock Type vs. *J*-Function

#### 3.3.1 Why Do We Need To Develop a *J*-Function?

It has been a practice in the geo-energy industry to predict water saturation based on the *J*-function, a normalized capillary pressure.^6,31^ Note: The capillary pressure curve is called the soil-water characteristic curve SWCC in civil and geotechnical engineering. Capillary pressure *P*_C_ is defined by pore radius *R*, rock wettability presented by contact angle θ, and water–oil interfacial tension σ, i.e., *P*_C_ = σ cos θ/*R*. Capillary radius *R* can be approached by the characteristic length:  that implies . The capillary rise *h* on a system with different densities, e.g., water ρ_w_ and oil ρ_o_, could elaborate the capillary pressure *P*_C_ = (ρ_w_– ρ_o_)*gh*. Thus, the dimensionless form of Leverett’s *J*-function is  or .

**Figure 6:**
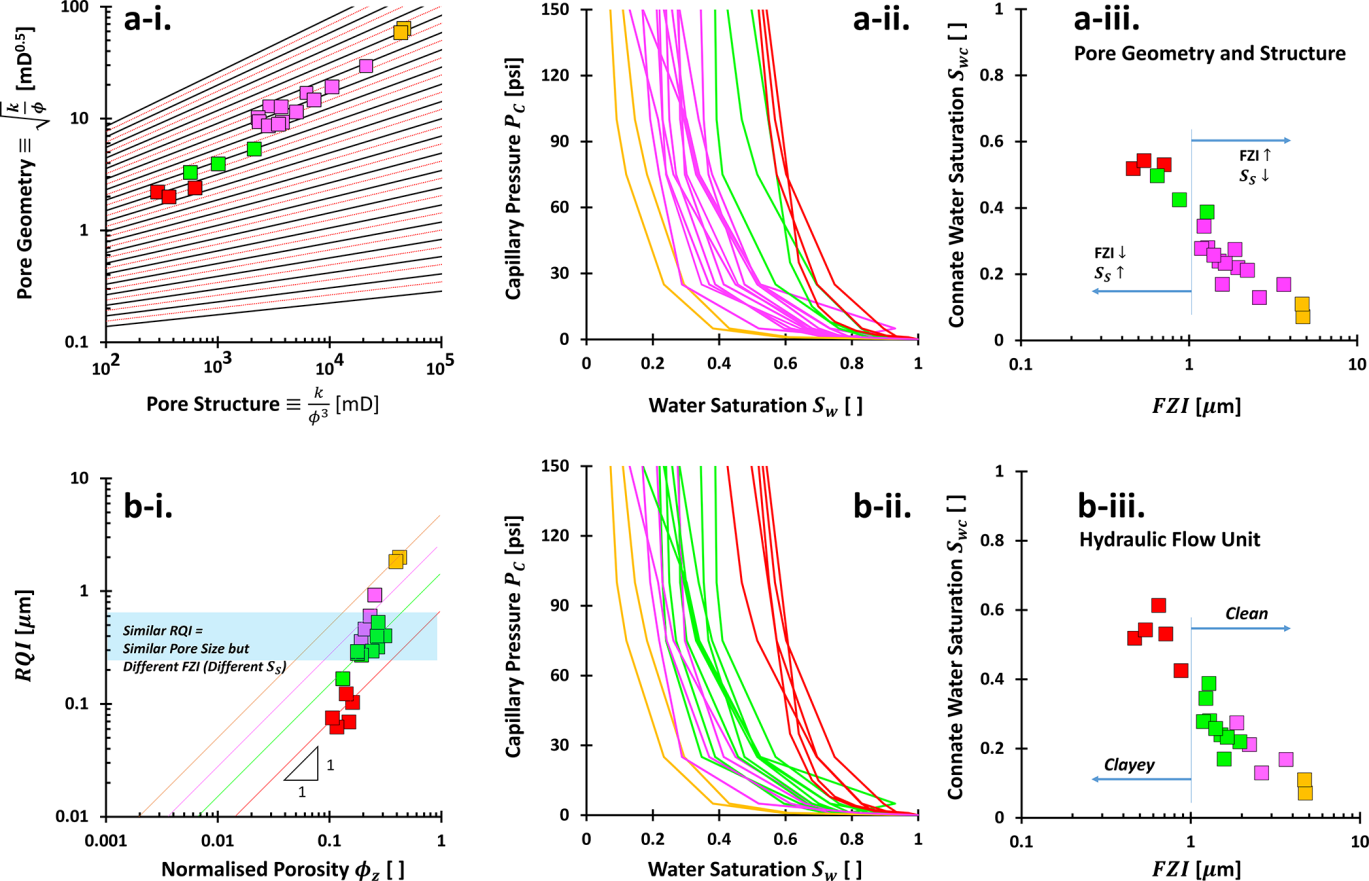
Capillary pressure grouping in sandstones. (a) Pore geometry and structure rock typing. Color codes hold several rock type units (RT #), called rock type group. The rock typing lines: black; boundary lines: red-dashed. (b) Hydraulic flow unit HFU. RQI stands for reservoir quality index. Color code symbolizes a single rock type equivalent to a flow zone indicator FZI unit. Data are from ref (67). Subfigure identities: (i) rock typing method, (ii) capillary pressure grouping, and (iii) connate water saturation vs FZI.

**Figure 7:**
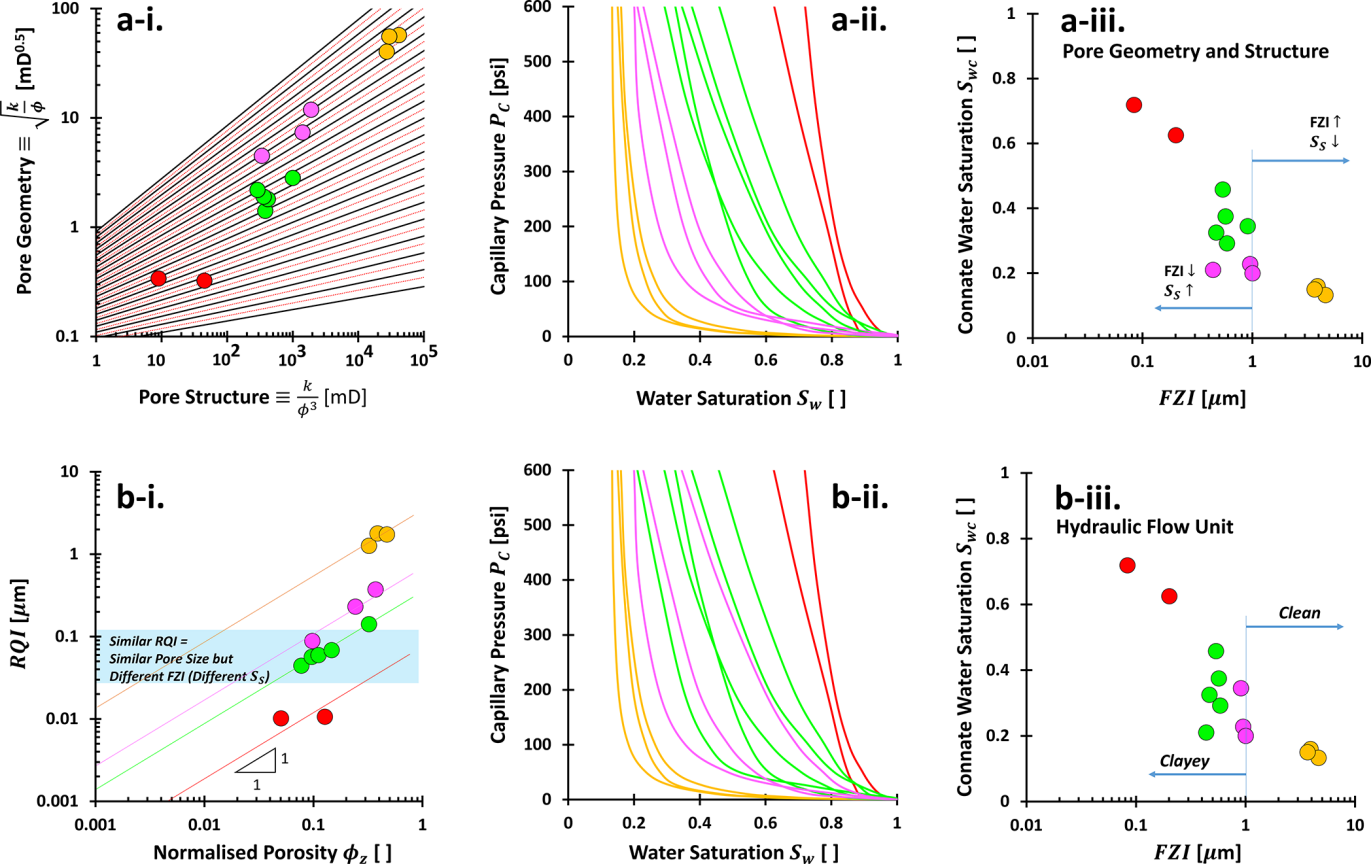
Capillary pressure grouping in carbonates. (a) Pore geometry and structure rock typing. Color codes hold several rock type units (RT #). The rock typing lines: black; boundary lines: red-dashed. (b) Hydraulic flow unit. Color code symbolizes a single rock type equivalent to a flow zone indicator FZI unit. RQI stands for reservoir quality index. Data source: X-Field, Indonesia. Subfigure identities: (i) rock typing method, (ii) capillary pressure grouping, and (iii) connate water saturation vs FZI.

The number of *J*-functions must be less than the capillary pressure curves (=number of data tested for special core analysis SCA). The results in Figures 6 and 7 point out that we need only four *J*-funtions (red, green, violet, and orange) declared by *J* = π_0_(*S*_w_– *S*_wi_)^−π_1_^ with each two positive fitting constants π_0_ and π_1_, also as a function of water saturation at arbitrary *S*_w_ and initial condition *S*_wi_. The capillary grouping (look at the color code) with PGS method actually includes a few rock type numbers, the closest ones. The representative *J*-function can be developed with a least-squares regression once all data with the same color code are already arranged into a single vector for each axis. By equating both *J* definitions between  and *J* = π_0_(*S*_w_– *S*_wi_)^−π_1_^, we can then predict the water saturation for the reservoir grid cell with
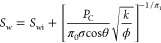
8

The prediction of water saturation is critical for computation of the initial oil-in-place.

#### 3.3.2 Why Does Not HFU Classify the Capillary Data Nicely?

Rocks within the same PGS group have similar *J*-functions, as stated in the Introduction section. We want to assert the robustness of PGS compared to the frequently used method, such as HFU (Figures 6a-i vs b-i and 7a-i vs b-i). PGS rock typing provides well-grouped capillary pressures on sandstone (Figure 6) and carbonate samples (Figure 7) compared with HFU method. The occurrence of overlapping capillary pressures has also been observed in the previous studies.^25,38^

The reservoir quality index RQI as the *y*-component in HFU method is defined by  in a unit of length dimension. Accordingly, this is used to deduce the pore size and tortuosity. The normalized porosity ϕ_*z*_ is defined as ϕ/(1 – ϕ) and is equivalent to the void ratio *e* in the geotechnical engineering community (*e* = *V*_p_/*V*_m_, pore-to-solid mineral volume). The relationship between RQI and ϕ_*z*_ results in the HFU rock typing named flow zone indicator FZI = RQI/ϕ_*z*_. Appendix C explains that HFU method is derived from the Kozeny–Carman equation and details the physical interpretations of the FZI unit, identified by
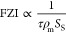
9

It is the deduction of the inverse values of tortuosity τ, mineral density ρ_m_, and specific surface area *S*_S_ (the derivation in eq C-6). HFU must adhere to the slope of one in the log–log plot because of the power of one in the normalized porosity ϕ_*z*_. The FZI emerges as the intercept or shifting constant, log FZI, in the log–log plot of HFU (equivalent to a slope in the linear–linear plot). Lower FZI values, for example, the red lines in Figures 6b-i and 7b-i, denote more tortuous, higher clay content, larger specific surface area, and/or denser materials. Clean sand would definitely be in the higher FZI groups, and clayey samples would be in the lower FZI group. However, a similar pore size *R*, manifested by similar RQI, can fall on the different FZI units. In fact, the capillary pressure *P*_C_ is intimately related to the reciprocal of pore size, *P*_C_ ∝ 1/*R*.

The “pore geometry” in PGS and RQI are actually the same but in different units; both are , but why are the overlapping capillary pressures frequently observable in HFU method, as proven in Figures 6a-ii, b-ii, and 7a-ii, b-ii? The *x*-component of HFU method is solely composed of porosity, whereas PGS method also captures permeability. The PGS’ *x*-component implicitly and indirectly represents the 1/(1 – ϕ)^2^ and all variables in the  (eqs 9 and C-6). Therefore, the “pore structure” in PGS: *k*/ϕ^3^ encompasses both quasi-porosity and FZI (tortuosity, density, and specific surface area).

Mathematically, PGS undergoes double processes: first, by quasi-HFU and then by line-based grouping. Physically, PGS classifies pore size and specific surface area simultaneously. Each PGS rock type clearly segregates pore size (Figure 3). Pore size mapping on the PGS plot proves this segregation, where the data span pore sizes of 3 orders of magnitude (soils from 1.7 to 250 μm in Figure 3a; sandstones 0.20–38.08 μm in Figure 3b). The color codes of pore size in carbonate data also progress from violet to red, i.e., increasing the 80th percentile pores d80 from 1.59 to 37.74 μm (Figure 3). Horizontal clustering is interpretable on the specific surface area distribution (Figure 4).

Moreover, each PGS rock type does not span a wide range of porosity ϕ (Figure 5) compared with that of HFU once the data appear on the conventional permeability-porosity plot (0.04 ≤ ϕ ≤ 0.22 in ref (31) ; 0.02 ≤ ϕ ≤ 0.83 in ref (68) ; 0.1 ≤ ϕ ≤ 0.4 in ref (69)). Each FZI or HFU unit inherently signifies a similar specific surface area, FZI ∝ 1/*S*_*S*_. However, a single value specific surface *S*_*S*_ in the Kozeny–Carman equation can span a broad range of permeability and porosity (Figures 1 and 5).

Thus, PGS works better than HFU method for capillary pressure grouping because PGS simultaneously clusters the pore size and specific surface area; meanwhile, HFU method simply classifies the specific surface area. Note: The variability of tortuosity τ and mineral density ρ_m_ values are minute. To this extent, we may conduct a future study that compares PGS and FZI* because previous studies conclude that FZI* exhibits better results on grouping relative permeability compared to conventional FZI and modified ones.^11,26,70^

### 3.4 Rock Type vs. Connate Water Saturation

In a dynamic reservoir simulation, the assignment of connate water saturation is used to predict the maximum nonwetting phase volume (e.g., oil, enriched gas). The saturation values are selected at the highest capillary pressure. We compare the connate water saturation and flow zone indicator FZI (Figures 6a-iii, b-iii, and 7a-iii, b-iii). PGS method groups the connate water in a vertical trend and each saturation range belongs to a different group of rock types. Overlaping connate water saturations are observable in different HFU rock types. This is the consequence of a better grouping in the capillary pressure by PGS method than those of HFU.

### 3.5 Rock Type vs. Resistivity

The electrical conductivity σ_T_ of the geomaterial is a total contribution from the mineral σ_m_, fluid σ_f_, and surface σ_S_ conductivity (derivation in Appendix D):
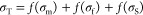
10

**Figure 8:**
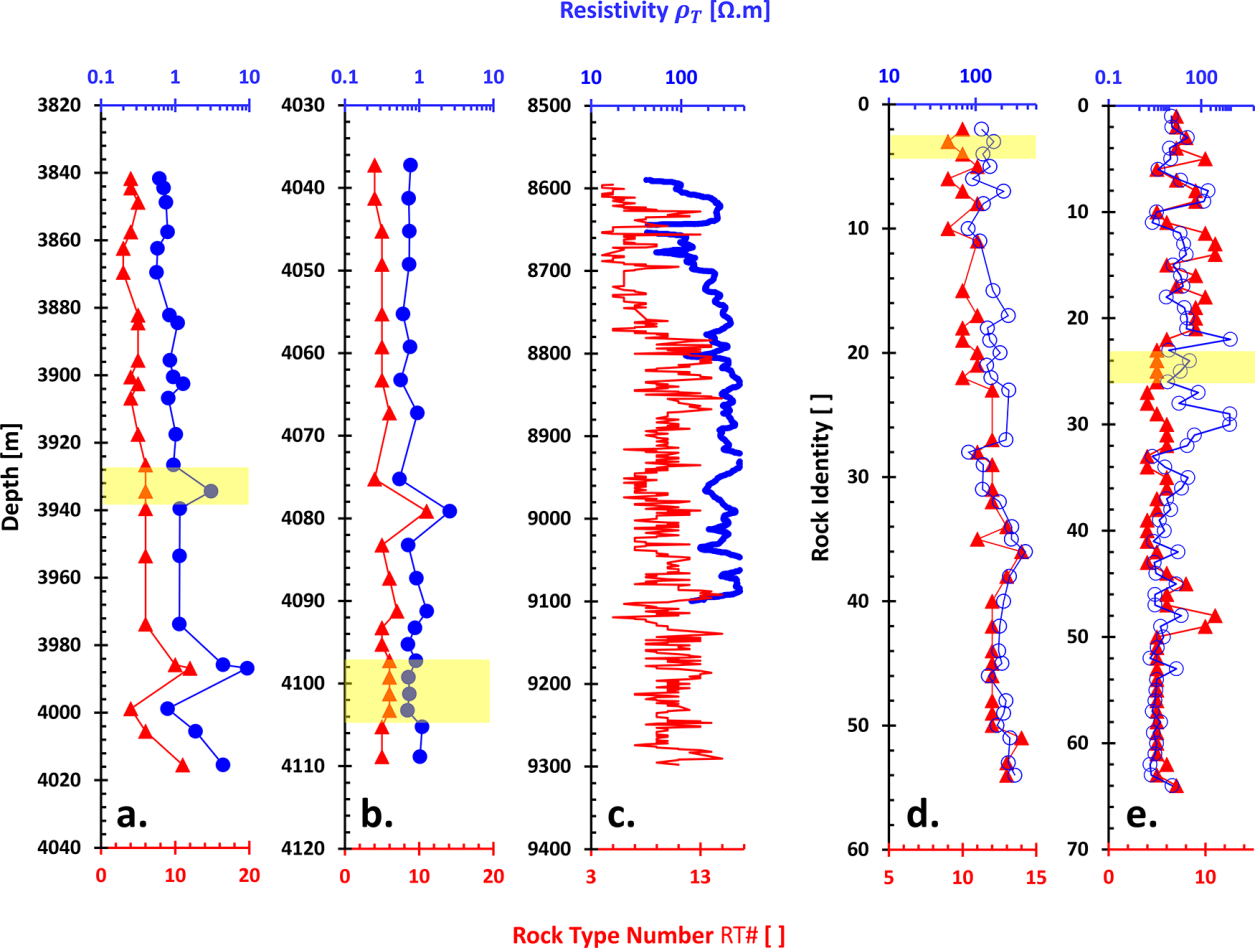
Resistivity vs. PGS rock type number: Spatial distributions. Data in (a) Well-A and (b) Well-BT2 are from the Volve field on the Norwegian continental shelf.^71^ (c) Sandstones. The resistivity data are derived from MSFL logging data from X-Field, Indonesia (ref (43) in ref (24)). (d) Tight sandstones. Presented resistivity data are normalized in the form of formation factor *F* (dimensionless), abstracted from ref (72). (e) Carbonate rocks. Data are from fields and out-crops in the Middle East, Australia, Italy, and the Bahamas.^73^ The yellow-highlighted areas are an anomaly, i.e., the rock type numbers are opposite the trend of resistivity. Note: Rock identity is equivalent to a hidden depth.

The inverted conductivity, which is equal to bulk or total resistivity ρ_T_, demonstrates a conformable fashion along the depth of the well with respect to the rock type number RT # (Figure 8). Lower resistivity ρ_T_ ↓ on more conductive materials displays smaller rock type numbers RT #↓ and vice versa.

However, the PGS rock type number RT # is inherently a rock classification based merely on hydraulic properties. Therefore, there is no direct causality relationship between RT # and resistivity. In fact, the PGS features control each resistivity ρ_T_ and rock type number independently. Table 2 summarizes the correlations among hydraulic properties, resistivity, and the PGS rock type number.

**Table 2:** Distinguished Variables among Hydraulic Properties that Control the Total Resistivity ρ_T_ and Rock Type Number RT # Relationships^a^

properties	direct causality	indirect correlations
tortuosity τ	τ ↑ → ρ_T_ ↑ and τ ↑ → RT # ↑	ρ_T_ ↑ → RT # ↑
pore size *R*	*R* ↑ → ρ_T_ ↓ and *R* ↑ → RT # ↓	ρ_T_ ↓ → RT # ↓
porosity ϕ	Liquids: ϕ ↑ → ρ_T_ ↓ and ϕ ↑ → RT # ↓	ρ_T_ ↓ → RT # ↓
	Gasses: ϕ ↑ → ρ_T_ ↑ and ϕ ↑ → RT # ↓	Anomaly: ρ_T_ ↑ → RT # ↓
specific surface area *S*_S_	*S*_S_ ↑ → ρ_T_ ↓ and *S*_S_ ↑ → RT # ↑	Anomaly: ρ_T_ ↓ → RT # ↑
permeability *k*eq A-9a:	ρ_T_ ≠ *f*(*k*) but ρ_T_ = *f*(τ, ϕ, *R*, *S*_S_) and *k* = *f*(τ, ϕ, *R*, *S*_S_), *k* ↑ → RT # ↓	Already described by τ, *R*, ϕ, and *S*_S_

a The upward arrow ↑ means increased values. The downward arrow ↓ means decreased values.

Below are the reasoning explanations that describe the correlation between resistivity and rock type number RT #:

- More resistive materials ρ_T_ ↑ can be due to more tortuous τ ≫ 1 pore paths. At the same time, τ ↑ affects the increased rock type number, RT # ↑.
- Less resistive materials ρ_T_ ↓ is prevalent in rocks with large pore size *R* ↑ due to improved ionic mobility *f*(σ_f_) ↑. The increased pore size implies a decreased rock type number, RT # ↓. The elaboration considers the relationship among pore size distribution, grain size distribution, and rock type numbers:^31,33,34^ larger pores or grains indicate a smaller rock type number RT # ↓. The larger pores (also associated with larger grains) would normally exhibit a high value of electrical conductivity σ_T_ ↑ (low resistivity ρ_T_ ↓). Therefore, a smaller rock type number corresponds to a lower resistivity sample.
- Less resistive materials ρ_T_ ↓ is also prevalent in high porosity ϕ ↑ rocks due to more spaces for the pore fluid, *f*(σ_f_) ↑. The increased porosity also implies a decreased rock type number, RT # ↓ (Figure 2a). A special case occurs if pores are gas filled or dry isolated because the increased isolated porosity hinders electrical conduction noted by the increased resistivity ρ_T_ ↑. Thus, an anomaly is accounted: ρ_T_ ↑ and RT # ↓ in unconnected pores.
- More conductive or less resistive ρ_T_ ↓ traits can be found in materials with high clay content *S*_S_ ↑ due to enhanced surface conduction *f*(σ_S_) ↑. However, the high specific surface materials shift the rock type toward a larger group RT # ↑. The correlation between ρ_T_ ↓ and RT # ↑ is opposite. Hence, we call this an anomaly. If we assess the pore size *R* and specific surface area *S*_S_ relationship:  in Appendix B, they are also contradictory, i.e., *R* ↑ → *S*_S_ ↓. Simultaneous large pore and high specific surface samples (*R* ↑ and *S*_S_ ↑) could be found in loose clays or unconsolidated high clay-content rocks. The pore size bounds the pore fluid contribution *f*(σ_f_) while the specific surface area governs the surface conduction effects *f*(σ_S_). The specific surface area prevails over the pore size in clayey rocks because *f*(σ_S_) ≫ *f*(σ_f_). The opposite case *f*(σ_S_) ≪ *f*(σ_f_) is prevalent in coarser, less clay-content materials, or geomaterials with a very conductive pore fluid σ_f_ > 1 S/m.^74−76^ These anomalies are observable in the yellow-highlighted zones in Figure 8a,d,e. The zone indicates a high calcite content (less clay), thereby increasing resistivity.
- A more conductive or less resistive ρ_T_ ↓ material can also be found because the metal inclusion enhances mineral conduction effects *f*(σ_m_) ↑. The metallic minerals do not contribute to the hydraulic properties. We can observe the anomaly of hindered resistivity in Figure 8b.
- What about permeability *k*? Permeability is measured at dynamic or under a flow process, occurring within a few seconds to minutes. Meanwhile, the resistivity measurement is conducted under a no-flow condition. The source of charge in wet geomaterials is mainly counterion clouds within the diffuse double layer and pore fluids, whose charge motion time frames are nano-to-microseconds.^49,75^ We can still measure resistivity on samples with closed pores (zero permeability), which does not allow fluid flow. A similar trend could also be found in NMR T2-relaxations: we can still measure water relaxation in closed pores, yet they may not work to predict permeability. The resistivity-permeability may display a strong correlation, but it is not a direct causation; yet, due to orchestrating factors among pore size *R*, specific surface area *S*_*S*_, and tortuosity τ.

**Figure 9:**
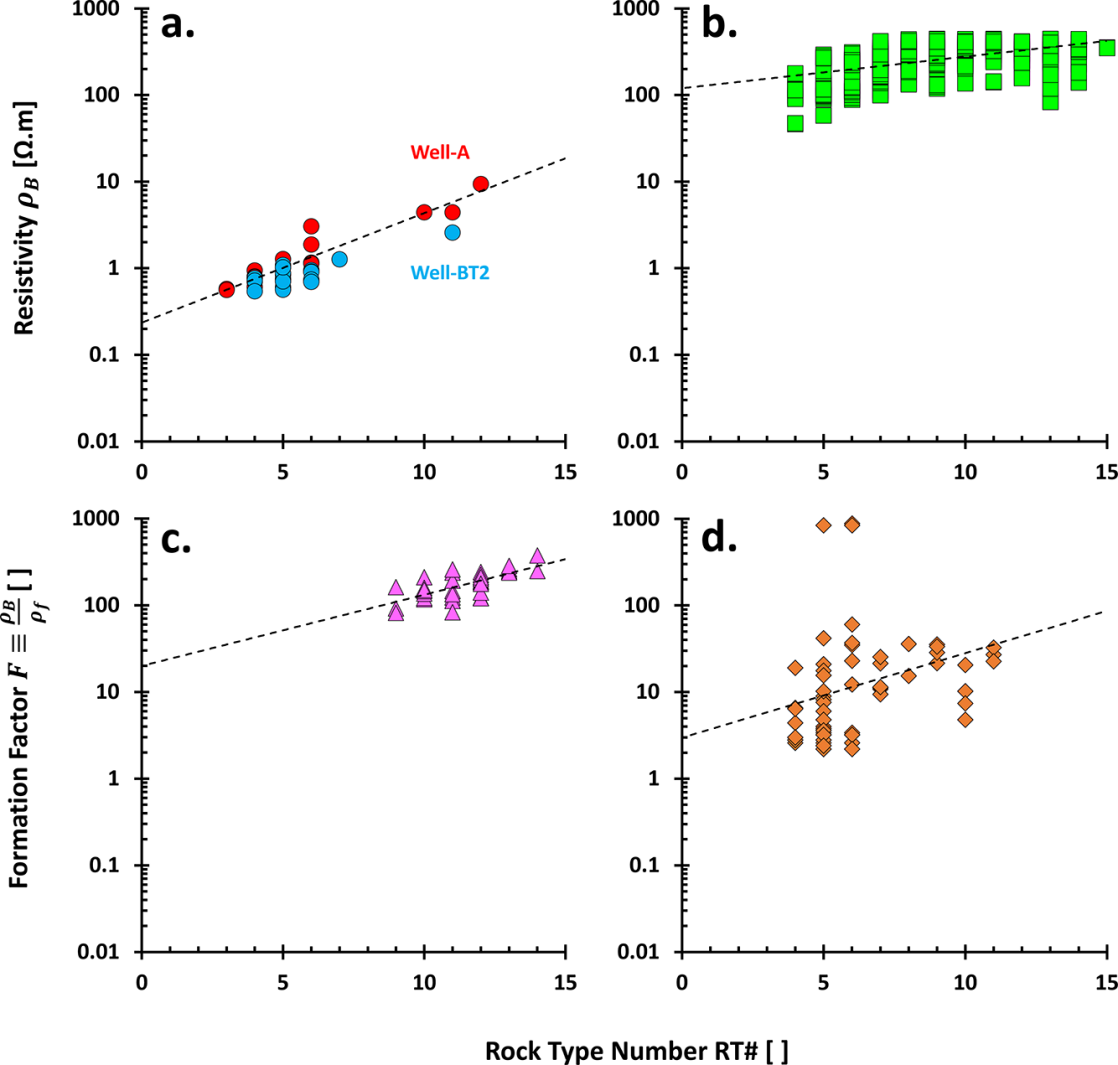
Resistivity vs. PGS rock type number: Cross-plots. (a) Volve.^71^ (b) Carbonate rocks.^24^ (c) Tight sandstones.^72^ (d) Carbonate rocks.^73^ Bulk resistivity ρ_B_ is equivalent to total resistivity ρ_T_ of the measured sample within the investigated area. Fitting lines are exponential functions that guide the eyes.

The cross-plot between bulk resistivity ρ_B_, equivalent to ρ_T_, versus rock type number RT# is generally quite good (Figure 9a–c) with an exponential trend: ρ_B_ ∝ exp (RT #) equivalent with log ρ_B_ ∝ RT#. However, they could also be sparse in certain carbonate rocks (Figure 9d). From the given well-logging data, such as resistivity, density, or neutron porosity, we could then predict the resistivity-based permeability *k̂*_ρ_ from the interpreted rock type using the following proposed steps (note: → and ← mean provides):

1. Resistivity → rock type # → the PGS constants *a* and *b*.
2. Density or neutron logs → porosity ϕ.
3. Predict the permeability *k̂*_ρ_ ← eq 1 is arranged to be , given *a*, *b*, and ϕ.

However, the limitation of these interpretations is that the lowest and highest rock type numbers RT # correspond to what minimum and maximum resistivity values. These values are case-dependent. These unclear boundaries could be solved by coring at selected depths to achieve match points or trends between bulk resistivity ρ_B_ and the RT #. For example, the data of well-A in Figure 9a emerge in an exponential trend of bulk resistivity ρ_B_ = 0.2353 exp (0.2914 × RT #) with *R*^2^ of more than 0.91.

### 3.6 Rock Type vs. Predicted Permeability

Permeability prediction assigns values in the static and dynamic geological models. The number of numerical model grids is much greater than the number of cored samples for routine core analysis RCA. The RCA provides measured permeability and porosity and subsequently the associated PGS grouping and constants {*a*, *b*}. The number of cored samples for RCA itself is much more than the number of samples tested for special core analysis SCA. Meanwhile, more continuous data could be obtained from well-logging. Summary about the number of data #: #_grid_ > #_logging_ > #_RCA_ > #_SCA_.

The predicted permeability *k̂* with PGS relies on both RCA and SCA data (complete derivation in Appendix E) and is given by
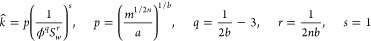
11

We see that *s* = 1,^35^ but there are previous studies that compute the *s* to be a rock type number-dependent, *s* = {*s* ≠ 1 | *f*(*a*, *b*)}.^42−46^ Note: This predicted permeability *k̂* is different from the resistivity-based permeability *k̂*_ρ_ that was discussed in the previous section.

The predicted permeability *k̂* = *f*(*a*, *b*, *m*, *n*) relies on {*a*, *b*} = *f*(*k*_RCA_, ϕ) and {*m*, *n*} = *f*(*k*_SCA_, *S*_w_). It is no wonder that the method consistently exhibits a good fit due to its recursive trait. We judge that this action is not an apple-to-apple appeal if we compare the predicted permeability between a recursive method (e.g., PGS) and forward calculation-based methods such as Tixer,^39^ Coates,^40^ and Timur.^41^ These forward equations are in explicit forms with predefined independent fitting constants

Furthermore, we do not even need prior PGS clustering if the permeability–porosity–saturation correlation is already in a good trend. A comparison should not be made between measured *k* and predicted permeability *k̂*; rather, the reservoir performance that is influenced mainly by permeability, e.g., flow rates. Alternatively, eq 11 predicts adjacent reservoir properties in nearby wells. Based on eq 8: *S*_w_ = *f*(*k*), therefore, the hydrocarbon-in-place can be used to check how good the prediction is.

## 4 Conclusions

This research has compiled thousands of experimental data from sandy, silty, clayey soils, sandstone, and carbonate rocks. We analyzed them extensively for the first time ever to interpret the Pore Geometry and Structure PGS rock typing, i.e., a method that plots permeability *k* and porosity ϕ in this style: . The pore geometry  elucidates the pore size *R* and tortuosity τ. Meanwhile, the pore structure *k*/ϕ^3^ deduces the effects of tortuosity τ and specific surface area *S*_*S*_. Notable observations are as follows:

- Experimentally measured hydraulic properties, such as permeability *k*, porosity ϕ, specific surface area *S*_S_, and pore size *R*, could match with the analytical model, the Kozeny–Carman equation, and span 12 orders of magnitude in permeability, porosity up to 0.9, 4 orders of magnitude in specific surface area, and pore radius ranges around 3 orders of magnitude.
- We have developed a physics-guided interpretation of the PGS rock typing: a lower rock type number RT # ↓ (e.g., close to clean sand) is due to corroborating factors such as increased permeability *k*, porosity ϕ, pore size *R*; or decreased tortuosity τ and specific surface area *S*_S_.
- The PGS rock typing’s exponent *b* = 0.5 (at *a* = 1) does not exclusively designate a cylindrical tube pore or a straight pore. However, it is a physically admissible value computed from the extreme hypothetical data, i.e., very porous materials with porosity ϕ ≥ 0.70 regardless of permeability, tortuosity, pore shape (geometric factor), pore size, and specific surface area.
- PGS method works better in clustering the capillary pressure and connate water saturation compared with Hydraulic Flow Unit HFU or also called Flow Zone Indicator FZI. PGS encompasses clustering based on both pore size and specific surface areas simultaneously, whereas HFU classifies rocks based on solely the specific surface area, FZI ∝ 1/*S*_S_. Therefore, PGS is a good tool for identifying the pore size distribution. Note: A better nomenclature is supposed to be the pore throat distribution because the data are derived from the capillary pressure.
- Causality and indirect correlations are distinguished among hydraulic properties, rock type number, and electrical resistivity. Hydraulic properties such as tortuosity τ, pore size *R*, porosity ϕ, and specific surface area *S*_S_ directly cause each of these independently: the electrical resistivity and the distribution of the PGS rock type. Therefore, the correlation between resistivity and rock type number is indirect. Generally, smaller rock type number corresponds to lower resistivity (higher electrical conductivity).
- Contradictory trends in the resistivity—rock type number could arise in some cases:
  i. Gas-saturated geomaterials: Increased gas-filled porosity and/or unconnected pores inhibit electrical charge transports.
  ii. Clayey rocks: Salient surface conduction effects *f*(σ_S_) over the pore fluid-induced conduction *f*(σ_f_). Specific surface area *S*_S_ and/or clay concentration (represented by a surface conduction factor Γ) dominantly govern the total conductivity of clayey materials instead of pore size and porosity.
  iii. Samples with high metallic mineral concentrations: Metal inclusions do not contribute to the hydraulic properties and PGS rock types, but they enhance electrical conductivity (reduced resistivity).
- The extended application of the resistivity—rock type number relationship is the advanced permeability *k̂*_ρ_ prediction from the well-logging data. Note: A few cores must be taken to delineate the minimum and maximum values between resistivity and rock type cluster.
- Permeability *k̂* prediction with the conventional PGS method manifests a recursive attribute and should not be compared with forward calculation predictors such as Tixier, Coates, and Timur. This prediction is more suitable for interpolating permeability in adjacent wells.

## Supporting Information Available

Detailed derivations of involved equations in this paper encompass: permeability derivation from Newton’s viscosity law and capillary bundled model; definition of specific surface area *S*_*S*_ ; Kozeny–Carman equation; electrical conductivity in wet porous media; and permeability fitting using PGS parameters. Also, effects of varied specific surface area *S*_*S*_ and tortuosity τ onto the PGS rock type; and the Matlab codes^77^ (PDF)
Data for main figures (XLSX)
Codes (ZIP)
