# Supplementary material for: On the Pore Geometry and Structure Rock Typing
Source: ACS Omega. 2024 Jul 25;9(32):34636–49. doi: 10.1021/acsomega.4c02879 (PMC11325524; doi:10.1021/acsomega.4c02879)
Supplement: Supplementary file 1 — ao4c02879_si_001.pdf [file ao4c02879_si_001.pdf]

SUPPLEMENTARY INFORMATION

On the pore geometry and structure rock typing

Published in ***ACS Omega***, July, 2024, DOI Link: https://doi.org/10.1021/acsomega.4c02879

AUTHORS:

Farizal Hakiki[a,b,c,*], Muhammad Nur Ali Akbar[d], Zaki Muttaqin[e]

*Corresponding author:

Full name: Farizal Hakiki Soemarsono, email: hakiki@nycu.edu.tw; farizal.hakiki@kaust.edu.sa

AFFILIATIONS:

[a] Griyanipun Tiang Sepah Kula, Malang 65153, Indonesia

[b] National Yang Ming Chiao Tung University, Disaster Prevention and Water Environment Research Center, Hsincu 30010, Taiwan

[c] National Yang Ming Chiao Tung University, Civil Engineering Department, Hsincu 30010, Taiwan

[d] Prores AS, Trondheim 7041, Norway

[e] (an independent researcher), Tangerang 15810, Indonesia

Supplementary Information emphasises: i) Detailed derivations (**Appendices A to E**) and ii) The effects of varied specific surface area S_S and tortuosity τ onto the Pore Geometry and Structure (PGS) Rock Type (**Appendix F**). Detailed derivations encompass: i) Permeability derivation from Newton's viscosity law and capillary bundled model (**Appendix A**), ii) Definition of specific surface area (**Appendix B**), iii) Kozeny-Carman equation (**Appendix C**), iv) Electrical conductivity in wet porous media modelled as parallel circuits (**Appendix D**), and v) Permeability fitting using PGS parameters (**Appendix E**).

Permeability in **Appendix F** is computed with Kozeny-Carman equation and porosity ranges 0.01-0.99. Matlab code to generate plots in **Appendix F** are available. We also provide all data for main figures that appear in our manuscript (Data for Figures.xlsx).

Repository link: https://doi.org/10.5281/zenodo.12817855

Repository at NYCU Dataverse: https://doi.org/10.57770/SCMDDF

TABLE OF CONTENTS

APPENDICES *3*

1. Appendix A: Permeability *3*

2. Appendix B: Specific surface area *6*

3. Appendix C: Kozeny-Carman equation *9*

4. Appendix D: Electrical conductivity *11*

5. Appendix E: PGS Permeability fitting *14*

6. Appendix F: Effects of specific surface area and tortuosity onto the PGS Rock Type *16*

6.1 Descriptions for the code: 18

6.2 Matlab codes*:* 18

REFERENCES *21*

APPENDICES

1. Appendix A: Permeability

Newton's Viscosity Law expresses that viscosity μ is a measure of its resistance to deforming at a certain shear rate dv/dr and the fluid deformation itself is due to a shear stress τ_D constituted by the drag force F_D working on a tangential surface S_p along the pipe with a length of ℓ:

$$\tau_D \equiv \frac{F_D}{S_p} = -\mu \frac{dv}{dr} \qquad \text{[A-1a]}$$

$$\frac{\Delta P . A_C}{2\pi r \ell} = -\mu \frac{dv}{dr} \qquad \text{[A-1b]}$$

$$\frac{\Delta P . \pi r^2}{2\pi r \ell} = -\mu \frac{dv}{dr} \qquad \text{[A-1c]}$$

The drag force F_D is quantifiable through a given pressure drop ΔP working on a cross-sectional area A_C. The tangential shear stress τ_D would resist the fluid flow and hinder velocity v across the radius of pipe r. The negative sign denotes the reduction of velocity with the growth of radius from the centre of the pipe. Note: vectors of v and r are perpendicular (Figure S 1).

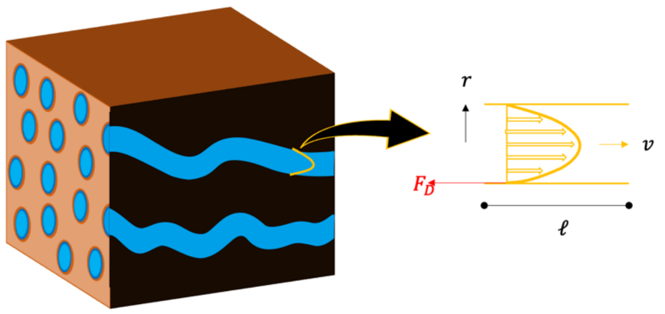

Figure S 1. Illustration of fluid flow in the pore. The drag force F_D on cylindrical pore with a length of ℓ. The distribution of velocity v decreases as the radius r reaches the edge of pipe at R.

Assign $v = 0$ at the pipe edge ($r = R$) and $v = v_{max}$ at the center o thef pipe ($r = 0$). Therefore, we have velocity v at an arbitrary r derived as follow

$$\int_R^r -\frac{\Delta P r}{2\ell\mu} dr = \int_0^v dv \qquad \text{[A-2a]}$$

$$-\frac{\Delta P}{2\ell\mu}\frac{(r^2 - R^2)}{2} = v \qquad \text{[A-2b]}$$

$$v = \frac{\Delta P}{4\mu\ell}(R^2 - r^2) \qquad \text{[A-2c]}$$

Compute the volumetric rate q passing through into a pipe with a crossectional area A_C:

$$q = \int v \ dA_C = \int_0^R \frac{\Delta P}{4\mu\ell} (R^2 - r^2)\, 2\pi r \, dr \qquad \text{[A-3a]}$$

$$q = \frac{\pi\Delta P}{2\mu\ell}\int_0^R R^2.r - r^3 \, dr \qquad \text{[A-3b]}$$

$$q = \frac{\pi\Delta P}{2\mu\ell}\left[\frac{R^2 r^2}{2} - \frac{r^4}{4}\right]_0^R \qquad \text{[A-3c]}$$

$$q = \frac{\pi\Delta P}{8\mu\ell}R^4 \qquad \text{[A-3d]}$$

We call Eq. A-3d as Hagen–Poiseuille equation. Tortuosity τ is the ratio between fluid path ℓ and the straight bulk length L, which has a dimensionless unit and typical values of

$$\tau \equiv \frac{\ell}{L} \geq 1 \qquad \text{[A-4]}$$

There are N numbers of bundled pipes that contribute to the total rate Q passing through the rock:

$$Q = Nq = N\left(\frac{\pi\Delta P}{8\mu\ell}R^4\right) \qquad \text{[A-5a]}$$

$$Q = \frac{N\pi\Delta P R^4}{8\mu\tau L} \quad \text{[A-5b]}$$

$$Q = \frac{N\pi R^4}{8\mu\tau}\frac{\Delta P}{L} \quad \text{[A-5c]}$$

$$Q = N\pi R^2\tau\frac{R^2}{8\mu\tau^2}\frac{dP}{dx} \quad \text{[A-5d]}$$

Note: Suppose $\frac{\Delta P}{L}$ is equivalent to a pressure gradient $\frac{dP}{dx}$. Recall the definition of porosity ϕ to define the bulk crossectional area A:

$$\phi = \frac{V_{pore}}{V_{bulk}} = \frac{N\pi R^2\ell}{A.L} = \frac{N\pi R^2}{A}.\tau \quad \text{[A-6a]}$$

$$N\pi R^2\tau = \phi A \quad \text{[A-6b]}$$

Arrange Eq. A-5d using A-6b, such that

$$Q = \phi A\frac{R^2}{8\mu\tau^2}\frac{dP}{dx} \rightarrow Q = \frac{\phi R^2}{8\tau^2}\frac{A}{\mu}\frac{dP}{dx} \quad \text{[A-7]}$$

Recall the definition of Darcy's equation:

$$Q = k\frac{A}{\mu}\frac{dP}{dx} \quad \text{[A-8]}$$

Then, equate the formulations in Eq. A-7 and A-8; therefore, the absolute permeability k equals to be

$$k \equiv \frac{\phi R^2}{8\tau^2} \quad \text{[A-9a]}$$

$$k \equiv \frac{\phi d^2}{32\tau^2} \quad \text{[A-9b]}$$

Note: The d is the pore diameter. The dimension of absolute permeability k is [L^2] with the unit of m^2, Darcy, or milli-Darcy mD.

We will derive the conversion of permeability in petroleum engineering k into geotechnical or civil engineering's permeability, known as hydraulic conductivity k_h, with the dimension of [L/T] or unit of cm/s or m/s. The total fluid velocity v_T in porous media is driven by the total head gradient $i \equiv dh/dx$ through a geomaterial with a hydraulic conductivity k_h:

$$v_T = k_h i \quad \text{[A-10]}$$

The studied liquid in geotechnical engineering is mainly water. So, it is not important to explicitly state the viscosity μ. Recall Q in Eq. A-8 to be

$$Q = v_T A \ \rightarrow v_T = \frac{k}{\mu}\frac{dP}{dx} \quad \text{[A-11a]}$$

Recall the definition of static pressure $P = \rho g h$ and the total head gradient $i = dh/dx$. Then, equate the petroleum engineering version on the left-hand-side with the geotechnical engineering version on the right-hand-side:

$$\frac{k}{\mu}\frac{dP}{dx} = k_h i \quad \text{[A-12a]}$$

$$\frac{k}{\mu}\rho g\frac{dh}{dx} = k_h\frac{dh}{dx} \quad \text{[A-12b]}$$

$$k = k_h\frac{\mu}{\rho g} \quad \text{[A-12c]}$$

Note that viscosity μ has a unit of mPa.s and dimension of [$ML^{-1}T^{-1}$]. Thus, we can convert the hydraulic conductivity k_h [LT^{-1}] into permeability k [L^2] using Eq. A-12c (unit in m^2). Then, we need the value of 0.9869233×10^{-12} m^2/D to convert into Darcy or 9.869233×10^{-16} m^2/mD (milli-Darcy).

2. Appendix B: Specific surface area

Volumetric specific surface area S_V [1/m] or [1/cm] is defined as

$$S_V = \frac{Inner\ Surface\ Area}{Bulk\ Volume} \quad \text{[B-1a]}$$

$$S_V = \frac{S}{V_T} \quad \text{[B-1b]}$$

$$S_V = \frac{N.2\pi R.\ell}{A.L} \quad \text{[B-1c]}$$

$$S_V = \frac{N2\pi R\tau}{A} \quad \text{[B-1d]}$$

$$S_V = 2\frac{N\pi R^2\tau}{A}\frac{1}{R} \quad \text{[B-1e]}$$

The substitution of $N\pi R^2\tau$ in Eq. A-6b into B-1e results in

$$S_V = 2\frac{\phi A}{A}\frac{1}{R} \quad \text{[B-2a]}$$

$$S_V = \frac{2\phi}{R} \quad \text{[B-2b]}$$

Gravimetric specific surface area S_S [m^2/g], also just called Specific surface with a known mineral density ρ_m is defined as follows:

$$S_S \equiv \frac{Inner\ Surface\ Area}{Solid\ Grain\ Mass} \quad \text{[B-3a]}$$

$$S_S = \frac{S}{\rho_m V_m} \quad \text{[B-3b]}$$

$$S_S = \frac{S}{\rho_m (V_T - V_P)} \quad \text{[B-3c]}$$

$$S_S = \frac{\frac{S}{V_T}}{\rho_m (1-\phi)} \quad \text{[B-3d]}$$

$$S_S = \frac{1}{1-\phi}\frac{S_V}{\rho_m} \quad \text{[B-3e]}$$

$$S_S = \frac{1}{1-\phi}\frac{2\phi}{\rho_m R}$$ [B-3f]

We evaluate a volumetric composition of mineral V_m that is obtained from total V_T and substracted by pore volume V_P. Substitute Eq. B-2b into B-3e in the process of derivation. Some works come up with the terminology of volumetric grain specific surface S_{V_g}, which is an analogue of S_S (Eq. B-3f):

$$S_{V_g} \equiv \frac{Inner\ Surface\ Area}{Solid\ Grain\ Volume}$$ [B-4a]

$$S_{V_g} = \frac{S}{V_m}$$ [B-4b]

$$S_{V_g} = \rho_m S_S$$ [B-4c]

$$S_{V_g} = \frac{1}{1-\phi}\frac{2\phi}{R}$$ [B-4d]

Next, let us derive another form of a specific surface S_S according to the physical structures of the mineral, for example, a long platy mineral as depicted in Figure S 2.

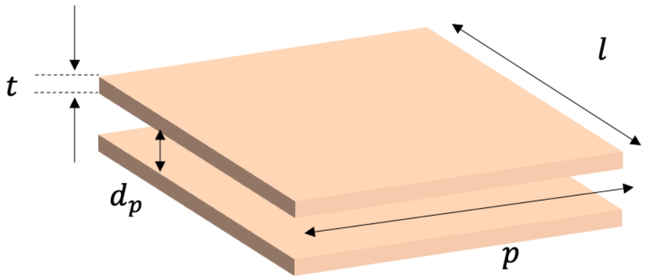

Figure S 2. Depiction of platy minerals to illustrate parallel clay.

Suppose $p > l \gg t$ which results in $1/t \gg 1/l > 1/p$, thereby the S_S is

$$S_S \equiv \frac{Surface\ Area}{Solid\ Grain\ Mass}$$ [B-5a]

$$S_S = \frac{2(pl + pt + lt)}{\rho_m(plt)} = \frac{2}{\rho_m}\left(\frac{1}{t} + \frac{1}{l} + \frac{1}{p}\right) \quad \text{[B-5b]}$$

$$S_S \approx \frac{2}{\rho_m t} \quad \text{[B-5c]}$$

Assume the structure is formed by a few platy minerals, i.e., parallel sheets. Therefore, the porosity and pore space d_p established in the parallel sheet emerge to be

$$\phi = \frac{d_p}{d_p + t} \rightarrow d_p = \frac{\phi}{1-\phi}t \quad \text{[B-6]}$$

Input the mineral thickness t from Eq. B-5c into Eq. B-6. Accordingly, the pore space in the parallel sheet d_p is then defined as

$$d_p = \frac{\phi}{1-\phi}\frac{2}{\rho_m S_S} \quad \text{[B-7]}$$

We recall B-3f in diameter variable $d = 2R$ (cylindrical pore) to be

$$d = \frac{\phi}{1-\phi}\frac{4}{\rho_m S_S} \quad \text{[B-8]}$$

See the comparison between Eqs. B-7 and B-8 designate parallel sheet vs cylindrical pores. We can generalise the factors of 2 (parallel sheet) or 4 (cylindrical) to be the geometrical factor α as in the pore diameter

$$d = \frac{\phi}{1-\phi}\frac{\alpha}{\rho_m S_S} \quad \text{[B-9]}$$

3. Appendix C: Kozeny-Carman equation

The substitution of $R = f(S_V)$ from Eq. B-2b, $R = f(S_S)$ from Eq. B-3f, and $d = f(S_S, \alpha)$ from Eq. B-9 into the definition of permeability k (Eq. A-9a) yields in the Kozeny-Carman equation in these forms:

$$k = \frac{\phi}{8\tau^2}\left(\frac{2\phi}{S_V}\right)^2 = \frac{\phi^3}{2\tau^2 S_V^2} \quad \text{[C-1]}$$

$$k = \frac{\phi}{8\tau^2}\left(\frac{1}{1-\phi}\frac{2\phi}{\rho_m S_S}\right)^2 = \frac{\phi^3}{2(1-\phi)^2}\frac{1}{\tau^2 \rho_m^2 S_S^2} \quad \text{[C-2]}$$

$$k = \frac{\phi}{32\tau^2}\left(\frac{\phi}{1-\phi}\frac{\alpha}{\rho_m S_S}\right)^2 = \frac{\phi^3}{32(1-\phi)^2}\frac{\alpha^2}{\tau^2 \rho_m^2 S_S^2} \quad \text{[C-3]}$$

We will compare the Kozeny-Carman equation in petroleum and geotechnical engineering. The equation in geotechnical engineering is presented as follows [1]:

$$k_h = \frac{e^3}{1+e}\frac{C_F g}{v_f}\frac{1}{\rho_m^2 S_S^2} \quad \text{[C-4a]}$$

Convert the hydraulic conductivity in geotechnical engineering k_h into petroleum engineering k (Eq. A-12c); alter the void ratio e into porosity ϕ with the relationship of $e = \phi/(1-\phi)$, and expand the kinematic fluid viscosity v_f to a dynamic fluid viscosity $\mu = \rho v_f$; those lead to

$$\frac{k\rho g}{\mu} = \frac{\phi^3/(1-\phi)^3}{1+\dfrac{\phi}{1-\phi}}\frac{C_F g}{\mu/\rho}\frac{1}{\rho_m^2 S_S^2} \quad \text{[C-4b]}$$

$$k = C_F \frac{\phi^3}{(1-\phi)^2}\frac{1}{\rho_m^2 S_S^2} \quad \text{[C-4c]}$$

Later, compare Eqs. C-2 and C-4c, which results in

$$C_F = \frac{1}{2\tau^2} \quad \text{[C-5]}$$

The value of pore topology constant C_F of 0.2 [1,2] suggests that the tortuosity of soil samples τ is around 1.58.

If we move a porosity variable ϕ into the left-hand side of Eq. C-2 and then perform square root operation, we get the concept of hydraulic flow unit HFU:

$$\sqrt{\frac{k}{\phi}} = \frac{\phi}{(1-\phi)} \times \frac{1}{\sqrt{2}\tau\rho_m S_S} \quad \text{[C-6a]}$$

$$RQI = \phi_Z \times FZI \quad \text{[C-6b]}$$

The HFU concept consists of Reservoir Quality Index RQI, normalised porosity ϕ_Z, and flow zone indicator FZI. This concept is just the way to plot where the FZI is not explicitly calculated from τ, ρ_m, and S_S but rather RQI/ϕ_Z. Note: the unit of k in Eq. C-6a is m^2.

4. Appendix D: Electrical conductivity

Suppose the value of one is expandable to a ratio of volume to volume:

$$1 = \frac{AL}{V_T} = \frac{A_f L_f}{V_f} = \frac{A_m L_m}{V_m} = \frac{A_S L_S}{V_S} \quad \text{[D-1]}$$

where the subscript T: total or bulk, f: fluid, m: mineral, and S: surface. Note that the volume is approached with a tube model with a cross-sectional area A and length L. Recall the definition of the resistance-resistivity R-ρ which is equivalent to conductance-conductivity G-σ:

$$R = \frac{L}{A}\rho \quad \text{[D-2a]}$$

$$G = \frac{A}{L}\sigma \quad \text{[D-2b]}$$

Electronic conductions in solid minerals, ionic conductions in pore fluid, and counter-ion transports due to mineral-fluid interactions can be modelled as a parallel circuit if each phenomenon is in the parallel orientation within the applied electrical field [3–5]. We use the basic equation (Eq. D-2) to model the parallel circuit among the fluid, mineral and surface components:

$$\frac{1}{R_T} = \frac{1}{R_f} + \frac{1}{R_m} + \frac{1}{R_S} \quad \text{[D-3a]}$$

$$G_T = G_f + G_m + G_S \quad \text{[D-3b]}$$

$$\sigma_T \frac{A}{L} = \sigma_f \frac{A_f}{L_f} + \sigma_m \frac{A_m}{L_m} + \sigma_S \frac{A_S}{L_S} \quad \text{[D-3c]}$$

$$\sigma_T = \sigma_f \frac{A_f}{A} \frac{L}{L_f} + \sigma_m \frac{A_m}{A} \frac{L}{L_m} + \sigma_S \frac{A_S}{A} \frac{L}{L_S} \quad \text{[D-3d]}$$

Suppose the tube length over the bulk length is defined as the tortuosity τ. The expression of surface-related cross-sectional area A_S is constructed from the diffuse double layer with the thickness of λ and covers along the pore perimeter $2\pi r$ (Figure S 3). Together with the definition of A_s, substitute Eq. D-1 into Eq. D-3d to obtain this set of equations:

$$\sigma_T = \sigma_f \frac{V_f}{V_T} \frac{L}{L_f} \frac{L}{L_f} + \sigma_m \frac{V_m}{V_T} \frac{L}{L_m} \frac{L}{L_m} + \sigma_S \frac{2\pi r \lambda}{A} \frac{L}{L_S} \quad \text{[D-4a]}$$

$$\sigma_T = \sigma_f \frac{V_f}{V_p} \frac{V_p}{V_T} \frac{1}{\tau_f^2} + \sigma_m (1-\phi) \frac{1}{\tau_m^2} + \sigma_S \frac{\lambda}{A} \frac{2\pi r L_S}{L_S} \frac{L}{L_S} \quad \text{[D-4b]}$$

$$\sigma_T = \sigma_f S_f \phi \frac{1}{\tau_f^2} + \sigma_m (1-\phi) \frac{1}{\tau_m^2} + \sigma_S \frac{\lambda}{A} S \frac{L}{L_S^2} \quad \text{[D-4c]}$$

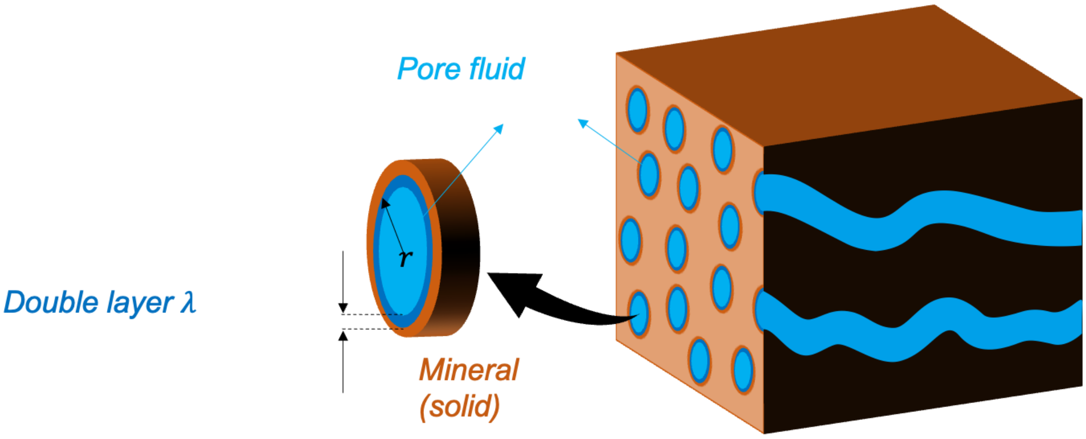

Figure S 3. Double layer λ emergence between the bulk pore fluid and solid surface. The thickness of $\lambda \ll r$ hence we can assume that r is the distance of solid surface to the centre of pore.

The internal surface S can be described with $S = S_S \rho_m V_m = S_S \rho_m (1-\phi) V_T$. Then, expand the $V_T = AL$; therefore,

$$\sigma_T = \sigma_f S_f \phi \frac{1}{\tau_f^2} + \sigma_m (1-\phi) \frac{1}{\tau_m^2} + \sigma_S \frac{\lambda}{A} S_S \rho_m (1-\phi) V_T \frac{L}{L_S^2} \quad \text{[D-5a]}$$

$$\sigma_T = \sigma_f S_f \phi \frac{1}{\tau_f^2} + \sigma_m (1-\phi) \frac{1}{\tau_m^2} + \sigma_S \frac{\lambda}{A} S_S \rho_m (1-\phi)(AL) \frac{L}{L_S^2} \quad \text{[D-5b]}$$

$$\sigma_T = \sigma_f S_f \phi \frac{1}{\tau_f^2} + \sigma_m (1-\phi) \frac{1}{\tau_m^2} + \sigma_S \lambda S_S \rho_m (1-\phi) \frac{1}{\tau_S^2} \quad \text{[D-5c]}$$

The thickness of diffuse double layer λ could be approached by the Debye-Huckel characteristic length [6]:

$$\lambda = \sqrt{\frac{\kappa' \varepsilon_0 k T}{2 N_A e^2 I}} \quad \text{[D-6a]}$$

$$I = \frac{1}{2} \sum_{i=1}^{N} c_i z_i^2 \quad \text{[D-6b]}$$

with the associated variables are bulk fluid relative permittivity κ', vacuum permittivity ε_0, Boltzmann constant k, temperature T, Avogadro number N_A, elementary charge e, ionic strength I, the concentration of each ion c_i, and valence number z_i.

We simplify the form of Eq. D-5c with assumed tortuosity $\tau_m = \tau_f = 1$, generally called as τ, and define a surface conduction factor $\Gamma = \sigma_S \lambda \rho_m / \tau_S^2$:

$$\sigma_T = \sigma_f S_f \phi + (\sigma_m + \Gamma S_S)(1-\phi) \quad \text{[D-7]}$$

In the clayey sample, the contribution of S_S is huge compared to σ_f and the mineral conductivity is minute, $\sigma_m \ll \sigma_T$.

The first term of Eq. D-5c prevails in a clean sample, which we name Archie's equation [7,8]:

$$\sigma_T = \sigma_f S_f \frac{\phi}{\tau^2}$$ [D-8a]

$$\sigma_T = \sigma_f S_f^{n_A} \frac{\phi^{m_A}}{a_t}$$ [D-8b]

and we use arbitrary positive constants m_A and n_A (Eq. D-8b) to fit the data and accommodate the surface conduction (2nd term of Eq. D-7). We call the squared-tortuosity τ^2 as the tortuosity factor $a_t > 0$. Saturated sample $S_f = 1$ alters Archie's equation to be

$$\sigma_T = \sigma_f \frac{\phi^{m_A}}{a_t}$$ [D-9]

Further, we define the formation factor F as

$$F \equiv \frac{\sigma_f}{\sigma_T} \equiv \frac{\rho_T}{\rho_f} = a_t \phi^{-m_A}$$ [D-10]

where $m_A > 0$. Note: electrical conductivity σ [S/m] is the reciprocal of resistivity ρ [Ω.m].

5. Appendix E: PGS Permeability fitting

The pore geometry and structure (PGS) concept is defined as the plot between $\sqrt{\frac{k}{\phi}}$ and $\frac{k}{\phi^3}$ that is connected by positive constants of a and b:

$$\sqrt{\frac{k}{\phi}} = a\left(\frac{k}{\phi^3}\right)^b$$ [E-1]

We will show the derivation of permeability prediction formulation using PGS constants a and b. The fitting method consists of two other constants m and n which correlate the water

saturation S_w and measured permeability k. The predicted permeability $\widehat{k}$ indeed uses a recursive method and non-explicitly forward model:

$$\widehat{k} = f(k, \phi, S_w) \qquad \text{[E-2]}$$

The following derivations detail formulations derived from routine core analysis RCA and special core analysis SCA data. The data derived from the SCA technique provides a negative slope $-n$ between the (irreducible) water saturation S_w and measured permeability k [9,10]:

$$S_w = mk^{-n} \rightarrow k = \left(\frac{S_w}{m}\right)^{-\frac{1}{n}} = \left(\frac{m}{S_w}\right)^{\frac{1}{n}} \qquad \text{[E-3]}$$

We set constants m and n as positive values. Substitution of Eq. E-3 into the left-hand side of Eq. E-1 results in

$$\sqrt{\frac{\left(\frac{m}{S_w}\right)^{\frac{1}{n}}}{\phi}} = a\left(\frac{k}{\phi^3}\right)^b \qquad \text{[E-4]}$$

The b-th root operation on both sides yields in

$$k = \frac{1}{a^{\frac{1}{b}}}\left[\frac{\left(\frac{m}{S_w}\right)^{\frac{1}{n}}}{\phi}\right]^{\frac{1}{2b}}\phi^3 = \frac{1}{a^{\frac{1}{b}}}\left(\frac{m}{S_w}\right)^{\frac{1}{2nb}}\phi^{3-\frac{1}{2b}} = \frac{1}{a^{\frac{1}{b}}}\left(\frac{m}{S_w}\right)^{\frac{1}{2nb}}\phi^{\frac{6b-1}{2b}}$$

$$= \frac{m^{\frac{1}{2nb}}}{a^{\frac{1}{b}}}\left(S_w^{-\frac{1}{n}}\phi^{6b-1}\right)^{\frac{1}{2b}} = \left(\frac{m^{\frac{1}{2n}}}{a}\right)^{\frac{1}{b}}\frac{\phi^{3-\frac{1}{2b}}}{S_w^{\frac{1}{2nb}}} \qquad \text{[E-5]}$$

Rearrange Eq. E-5 to state the predicted permeability $\widehat{k}$ explicitly:

$$\hat{k} = p\left(\frac{1}{\phi^q S_w^r}\right)^s; \quad p = \left(\frac{m^{\frac{1}{2n}}}{a}\right)^{\frac{1}{b}}; \quad q = \frac{1}{2b} - 3; \quad r = \frac{1}{2nb}; \quad s = 1 \qquad \text{[E-6]}$$

Note: the constants $\{a, b\}$ are from RCA data, while $\{m, n\}$ are from SCA tests.

6. Appendix F: Effects of specific surface area and tortuosity onto the PGS Rock Type

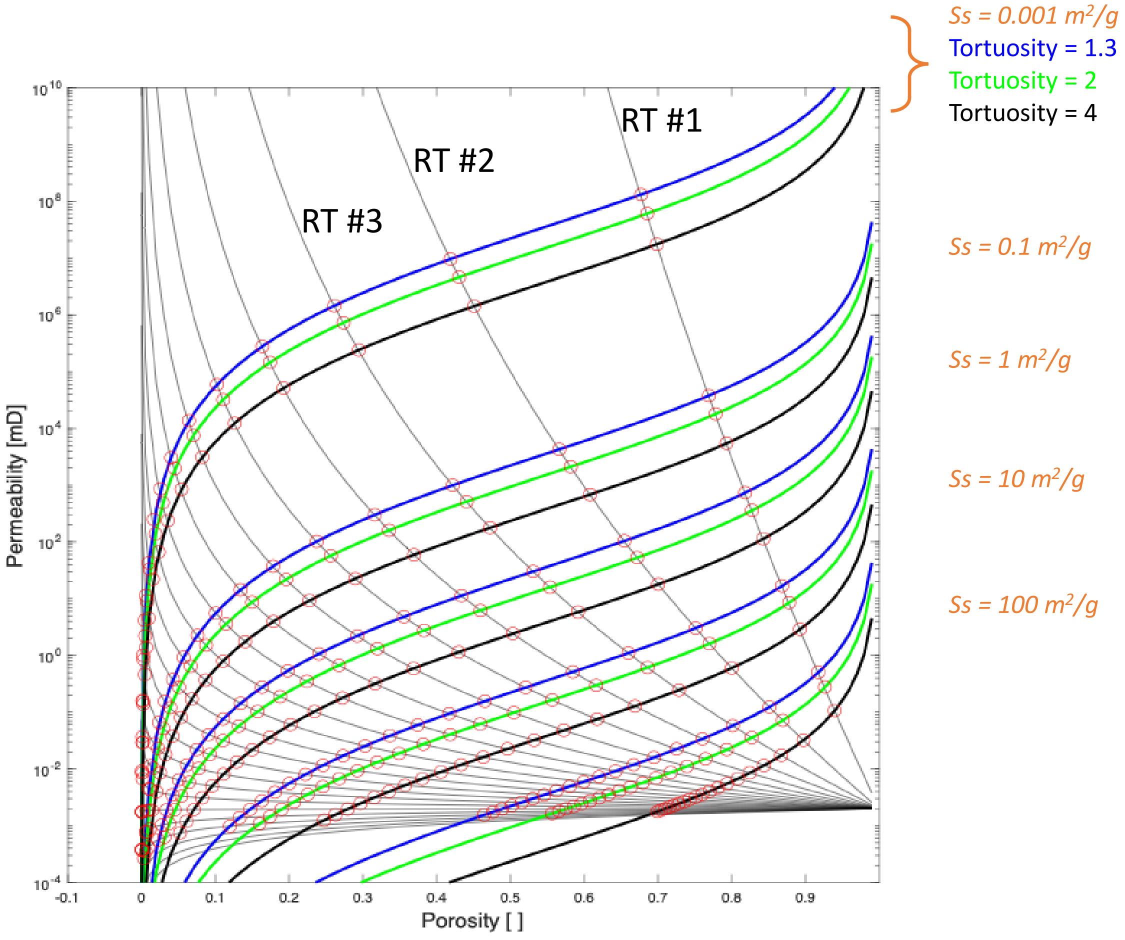

Figure S 4. Conventional permeability-porosity cross-plot with known PGS rock type RT lines. PGS stands for the Pore Geometry and Structure. Permeability is computed from the Kozeny-Carman equations with porosity from 0.0001 to 0.99. Circles are to show the intersection points. Symbol of Ss for specific surface area.

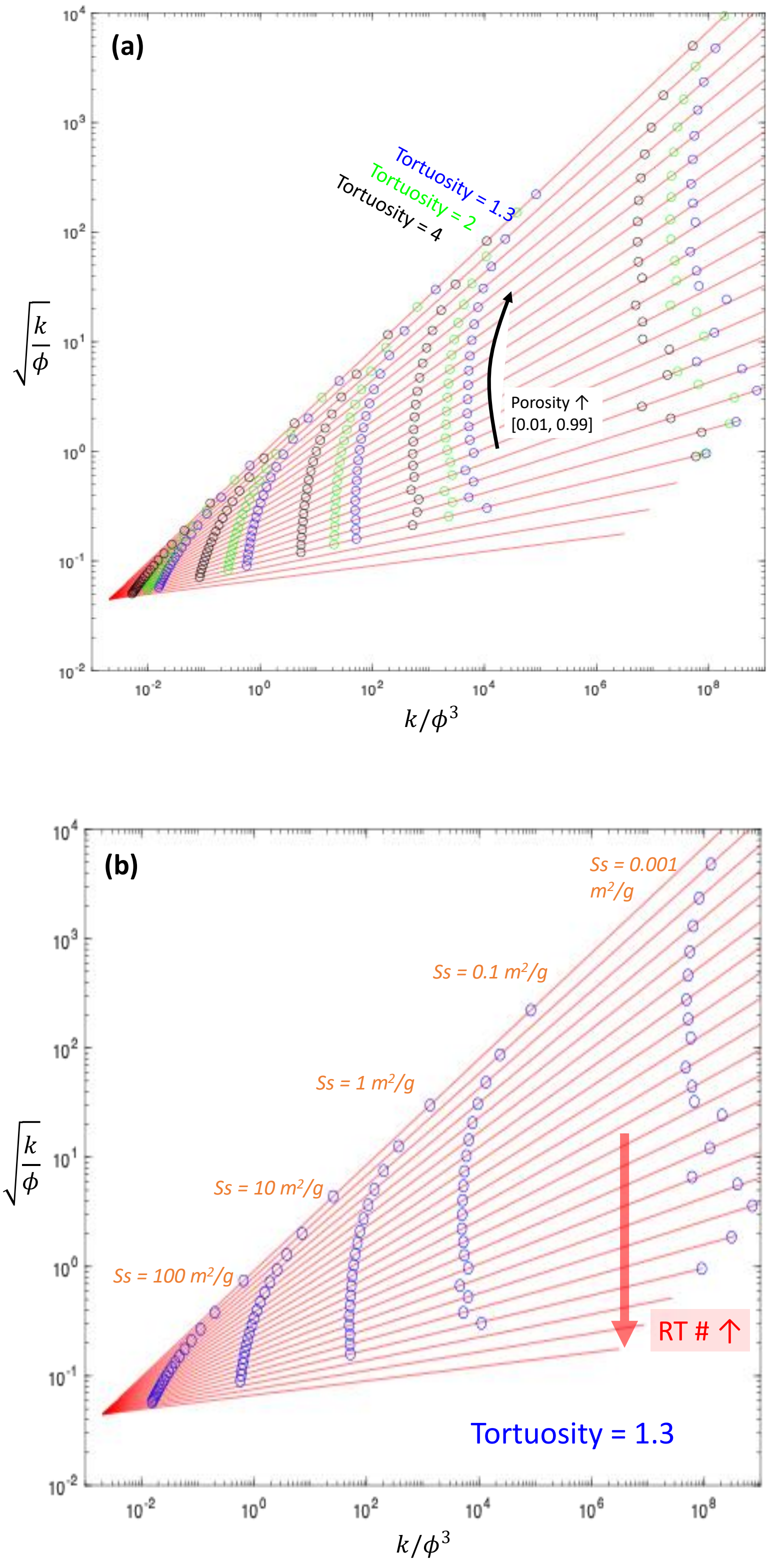

Figure S 5. PGS plot with known tortuosity and specific surface area. Each circle is from the intersection points between lines in Figure S 4. Part (a) showing all typical tortuosities. Part (b) emphasizes the distribution of specific surface area. RT # means Rock Type Number. Symbol of Ss for specific surface area.

6.1 Descriptions for the code:

Permeability *k from Kozeny-Carman*

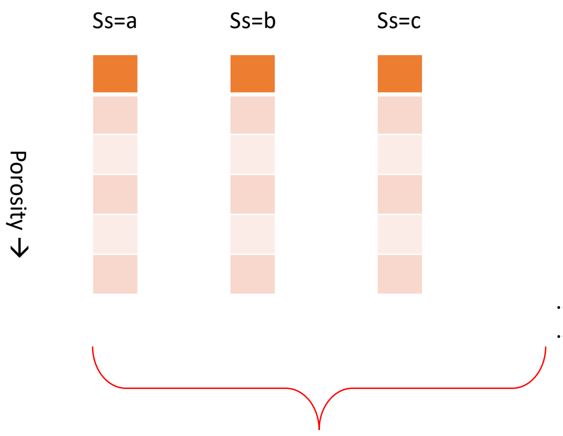

… number of case = (number of Ss) X (number of Tortuosity)
… N = NSs X NTor

Each NSs columns of k, distinct Tortuosity

Note: Ss = Specific surface area [m^2/g]

Permeability k_{RT} *from Rock-Type*

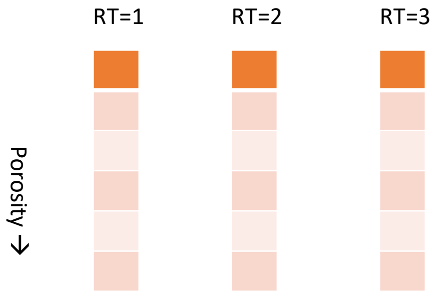

… number of Rock Type: NRT

Permeability k_i *and* Porosity ϕ_i *which intersected between* k *and* k_{RT}

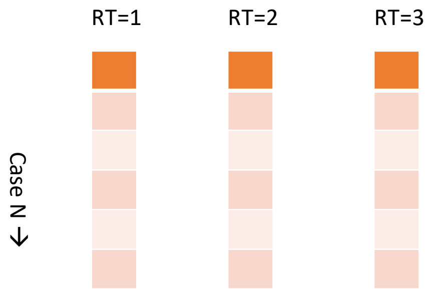

… number of Rock Type: NRT

6.2 Matlab codes:

```
% Written by Farizal Hakiki Soemarsono
% Email: farizal.hakiki@kaust.edu.sa
% Updated: 2022 November 22
```

```
close all; clear all; clc;
Phi = 0.01:0.01:0.99;
Por = [1e-4 1e-3 0.005 Phi]; Por = Por';
tor = [1.3 2 4]; % dimensionless
ntr = length(tor);
rho = 2.6; % g/cc
Ss  = [0.001 0.1 1 10 100]; % m^2/g
nSs = length(Ss);
c1  = 1e-12; % m6/cm6;
c0  = 9.869233e-16; % m2/mD
col = ['b', 'g', 'k']; %colour

NRT  = 22; % Number of RockType you want
for i=1:NRT
    if i==1;
        b(i)  = 0.4850;
        bB(i) = 0.4771;
        a(i)  = 0.002.^(0.5-b(i));
        aB(i) = 0.002.^(0.5-bB(i));

    else
        b(i)  = b(i-1)-0.02;
        bB(i) = bB(i-1)-0.02;

        a(i)  = 0.002.^(0.5-b(i));
        aB(i) = 0.002.^(0.5-bB(i));
    end
end

figure(1)
for i=1:NRT
    x(:,i)    = a(i).^2.*Por.^(1-6*b(i));
    kRT(:,i)  = nthroot(x(:,i),1-2*b(i));
    semilogy(Por,kRT(:,i),'k'); hold on %Rock Type

%     xB (:,i)  = aB(i).^2.*Por.^(1-6*bB(i));
%     kRTB(:,i) = nthroot(xB(:,i),1-2*bB(i));
%     semilogy(Por,kRTB(:,i),'-.r'); hold on %Rock Type Boundary
end

%Kozeny-Carman
i=1;
for j=1:ntr
    for jj=1:nSs
        k(:,i)   =  c1/c0.*0.5.*(Por.^3)./((1-Por).*tor(j).*rho.*Ss(jj)).^2;
        semilogy(Por,k(:,i),col(j),LineWidth=2); hold on
        xlim([-0.1 1]); ylim([1e-4 1e10]);
        xlabel('Porosity [ ]','FontSize',15); ylabel('Permeability [mD]','FontSize',15);
        i=i+1;
        %Each column = distinct Specific surface
        %Each 5 columns = distinct tortuosity, 5 = nSs
    end
end
```

```
[mk nk] = size(k);
%We set the number of rock type to 19, RT# = column
nRT = 19;
%Row = 1 to 15, each case for each Ss and tortuosity, nk = NRT*nSs cases
for j=1:nRT
    %Each RT at j
    for kk=1:nk
        %Each case at kk
        [xi,yi] = polyxpoly(Por,k(:,kk),Por,kRT(:,j)); hold on

mapshow(xi(xi==max(xi)),yi(xi==max(xi)),'DisplayType','point','Marker','o',Color='m',MarkerSize=9);
        Pori(kk,j) = xi(xi==max(xi));
        ki(kk,j)   = yi(xi==max(xi));
    end
end
ax = gca;
ax.FontSize = 18;

figure(2)
%PGS Plot
%Data from Kozeny-Carman
i=1;
for j=1:ntr
    for jj=1:nSs
        Y(:,i)  = sqrt(k(:,i)./Por);
        X(:,i)  = k(:,i)./(1-Por).^3;
        loglog(X(:,i),Y(:,i),col(j)); hold on
        i=i+1;
    end
end

%Data from RT, Rock Typing Line
for i=1:NRT
    YRT = sqrt(kRT(:,i)./Por);
    XRT = kRT(:,i)./(Por.^3);
    loglog(XRT,YRT,'r'),; hold on
end
xlim([1e-3 1e9]); ylim([1e-2 1e4]);
ylabel('sqrt(k/\phi)','FontSize',15); xlabel('k/\phi^3','FontSize',15);
ax = gca;
ax.FontSize = 18;

figure(3)
%Data from RT
%Rock Typing Line
for i=1:NRT
    YRT = sqrt(kRT(:,i)./Por);
    XRT = kRT(:,i)./(Por.^3);
    loglog(XRT,YRT,'r'),; hold on
end

%Each colours = Distinct Tortuosity
Yi = sqrt(ki./Pori);
Xi = ki./(Pori.^3);
coltor = ['ob', 'og', 'ok'];
```

```
% L=1:nSs;
% for j=1:ntr
%     loglog(Xi(L,:),Yi(L,:),coltor(j)); hold on
%     L=L+nSs;
% end
loglog(Xi(1:5,:),Yi(1:5,:),'ob'); hold on
loglog(Xi(6:10,:),Yi(6:10,:),'og'); hold on
loglog(Xi(11:15,:),Yi(11:15,:),'ok'); hold on


xlim([1e-3 1e9]); ylim([1e-2 1e4]);
ylabel('sqrt(k/\phi)','FontSize',15); xlabel('k/\phi^3','FontSize',15);
ax = gca; ax.FontSize = 18;
```

REFERENCES

(1) Taylor, D. W. Fundamental of Soil Mechanics. **1948**. https://doi.org/https://www.amazon.com/Fundamentals-Soil-Mechanics-Donald-Taylor/dp/B000IN5E3W.

(2) Ren, X. W.; Santamarina, J. C. The Hydraulic Conductivity of Sediments: A Pore Size Perspective. *Eng Geol* **2018**, *233*, 48–54. https://doi.org/https://doi.org/10.1016/j.enggeo.2017.11.022.

(3) Pfannkuch, H. On the Correlation of Electrical Conductivity Properties of Porous Systems with Viscous Flow Transport Coefficients. In *Developments in Soil Science*; IAHR, Ed.; Elsevier, 1972; Vol. 2, pp 42–54. https://doi.org/https://doi.org/10.1016/S0166-2481(08)70527-0.

(4) Klein, K. A.; Santamarina, J. C. Electrical Conductivity in Soils: Underlying Phenomena. *J Environ Eng Geophys* **2003**, *8* (4), 263–273. https://doi.org/10.4133/JEEG8.4.263.

(5) Choo, H.; Burns, S. E. Review of Archie's Equation through Theoretical Derivation and Experimental Study on Uncoated and Hematite Coated Soils. *J Appl Geophy* **2014**, *105*, 225–234. https://doi.org/10.1016/j.jappgeo.2014.03.024.

(6) Ridwan, M. G.; Kamil, M. I.; Sanmurjana, M.; Dehgati, A. M.; Permadi, P.; Marhaendrajana, T.; Hakiki, F. Low Salinity Waterflooding: Surface Roughening and Pore Size Alteration Implications. *J Pet Sci Eng* **2020**, *195*, 107868. https://doi.org/https://doi.org/10.1016/j.petrol.2020.107868.

(7) Archie, G. E. The Electrical Resistivity Log as an Aid in Determining Some Reservoir Characteristics. *Transactions of the AIME* **1942**, *146* (01), 54–62. https://doi.org/10.2118/942054-G.

(8) Archie, G. E. Introduction to Petrophysics of Reservoir Rocks. *Am Assoc Pet Geol Bull* **1950**, *34*, 943–961.

(9) Khoironi, B. Study of Microfactures Effect on Rock Typing and Well Logs Reading in Carbonate Rock. **2013**. https://doi.org/https://webpac.lib.itb.ac.id/find/goMatch?q=khoiron&bFind=author.

(10) Wibowo, A. S.; Permadi, P. A Type Curve for Carbonates Rock Typing. In *All Days*; IPTC, 2013. https://doi.org/10.2523/IPTC-16663-MS.
