# Supplementary material for: On the Pore Geometry and Structure Rock Typing
Source: ACS Omega. 2024 Jul 25;9(32):34636–49. doi: 10.1021/acsomega.4c02879 (PMC11325524; doi:10.1021/acsomega.4c02879)
Supplement: Supplementary file 3 — ao4c02879_si_003.zip [file ao4c02879_si_003.zip › Code/PGS_Code.pptx]

## Slide 1
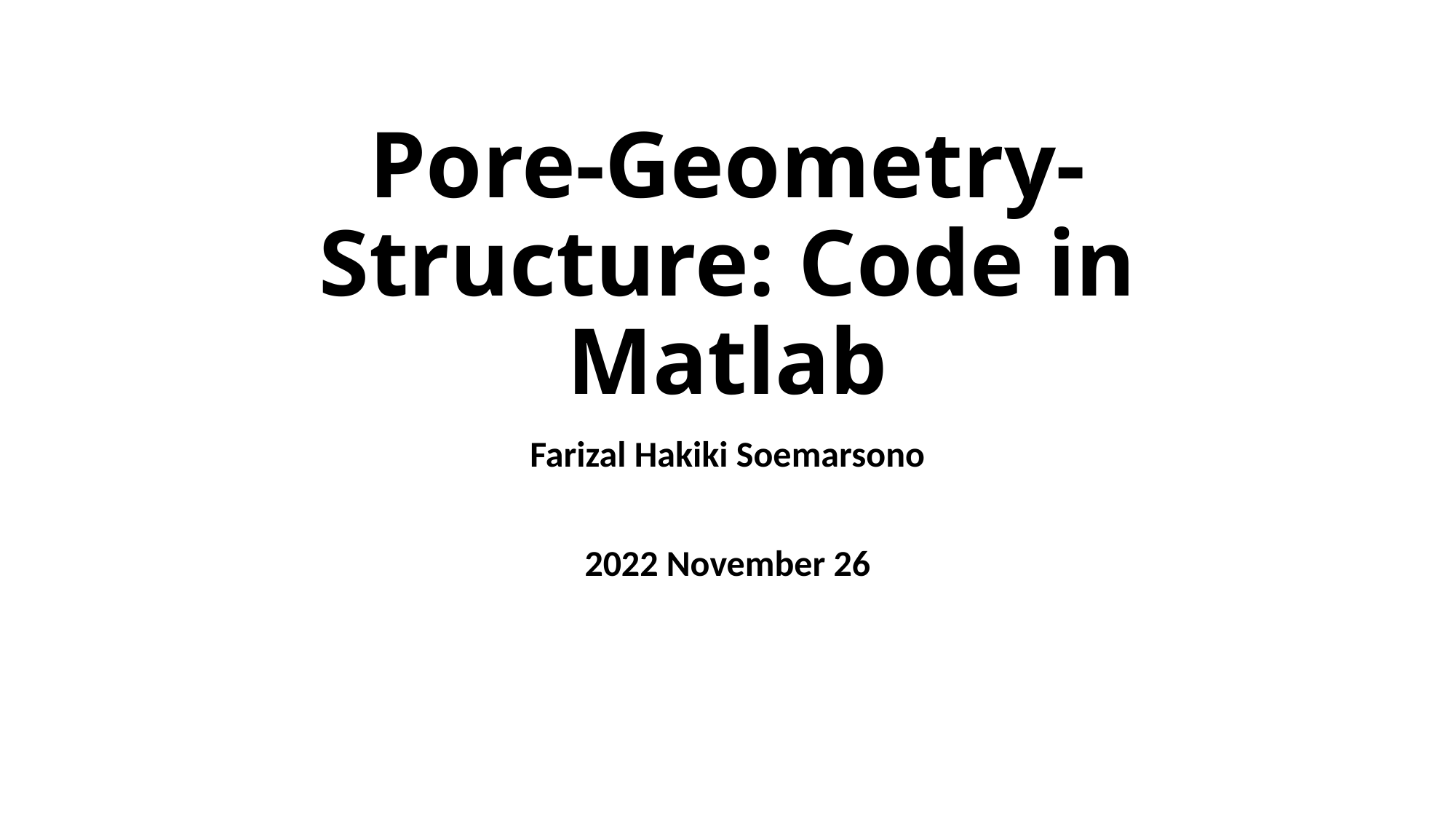

# Pore-Geometry-Structure: Code in Matlab
Farizal Hakiki Soemarsono
2022 November 26

## Slide 2
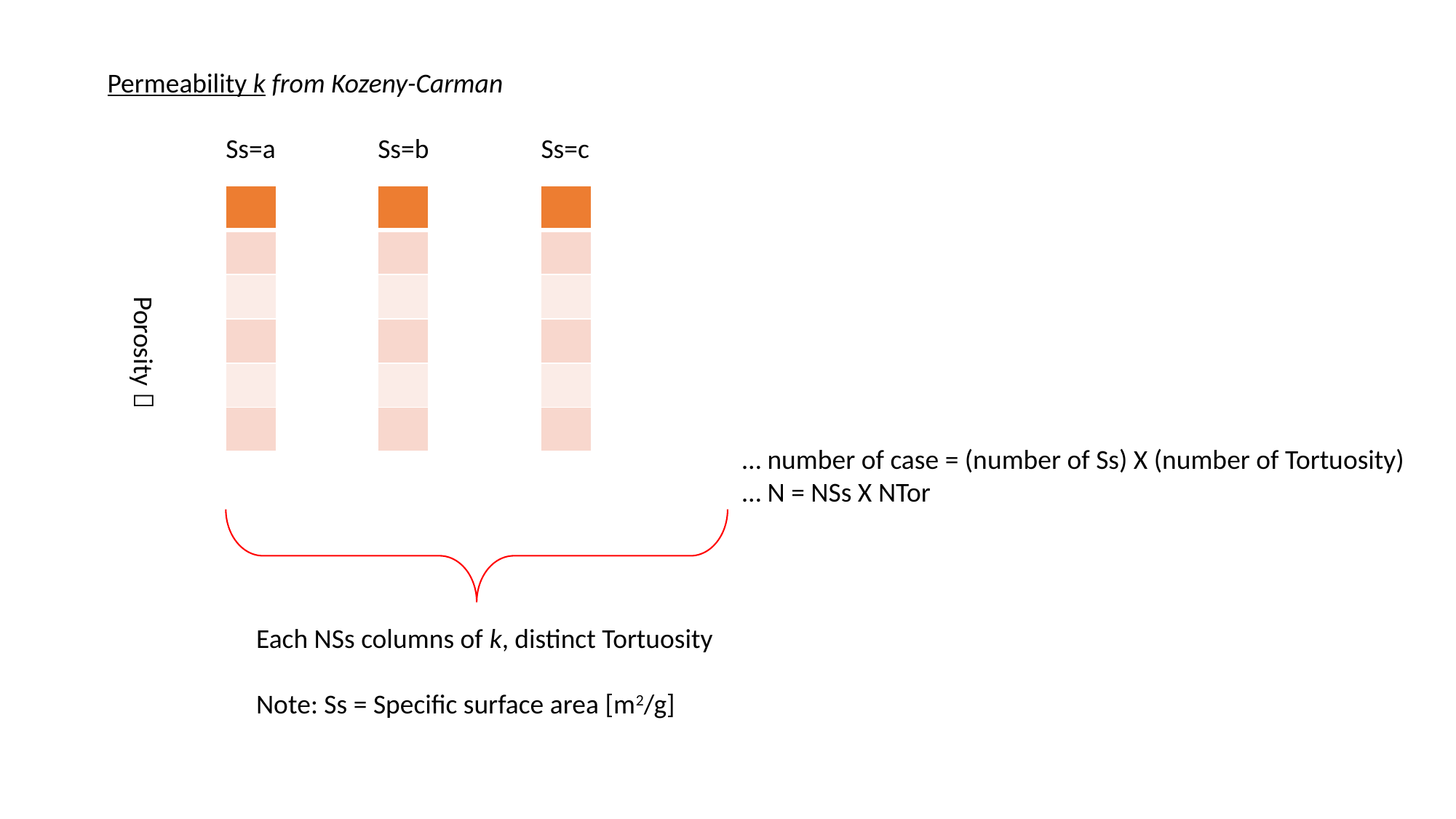

Permeability k from Kozeny-Carman
Ss=a
Ss=b
Ss=c
| |
| --- |
| |
| |
| |
| |
| |
| |
| --- |
| |
| |
| |
| |
| |
| |
| --- |
| |
| |
| |
| |
| |
Porosity 
… number of case = (number of Ss) X (number of Tortuosity)
… N = NSs X NTor
Each NSs columns of k, distinct Tortuosity
Note: Ss = Specific surface area [m2/g]

## Slide 3
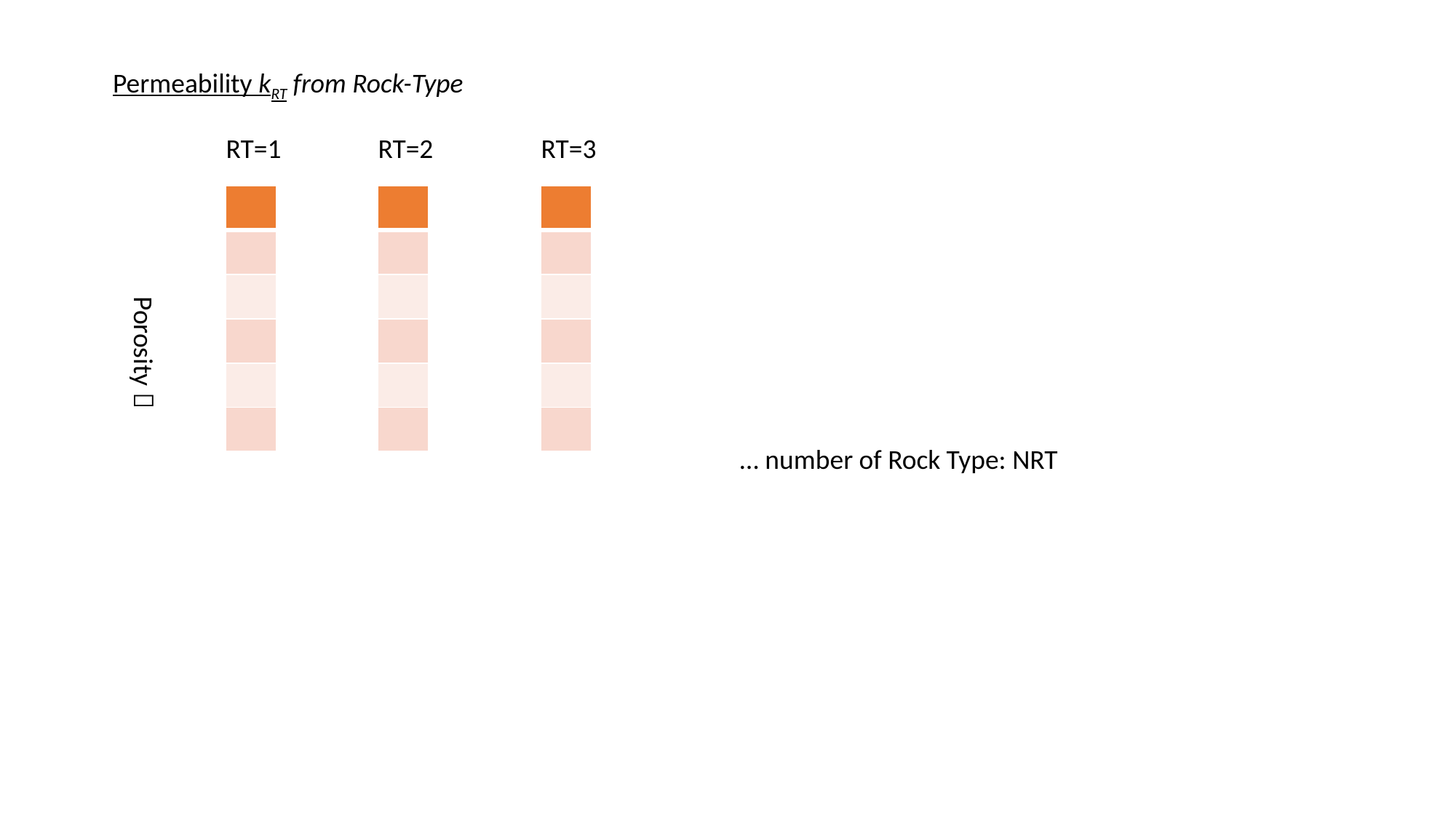

Permeability kRT from Rock-Type
RT=1
RT=2
RT=3
| |
| --- |
| |
| |
| |
| |
| |
| |
| --- |
| |
| |
| |
| |
| |
| |
| --- |
| |
| |
| |
| |
| |
Porosity 
… number of Rock Type: NRT

## Slide 4
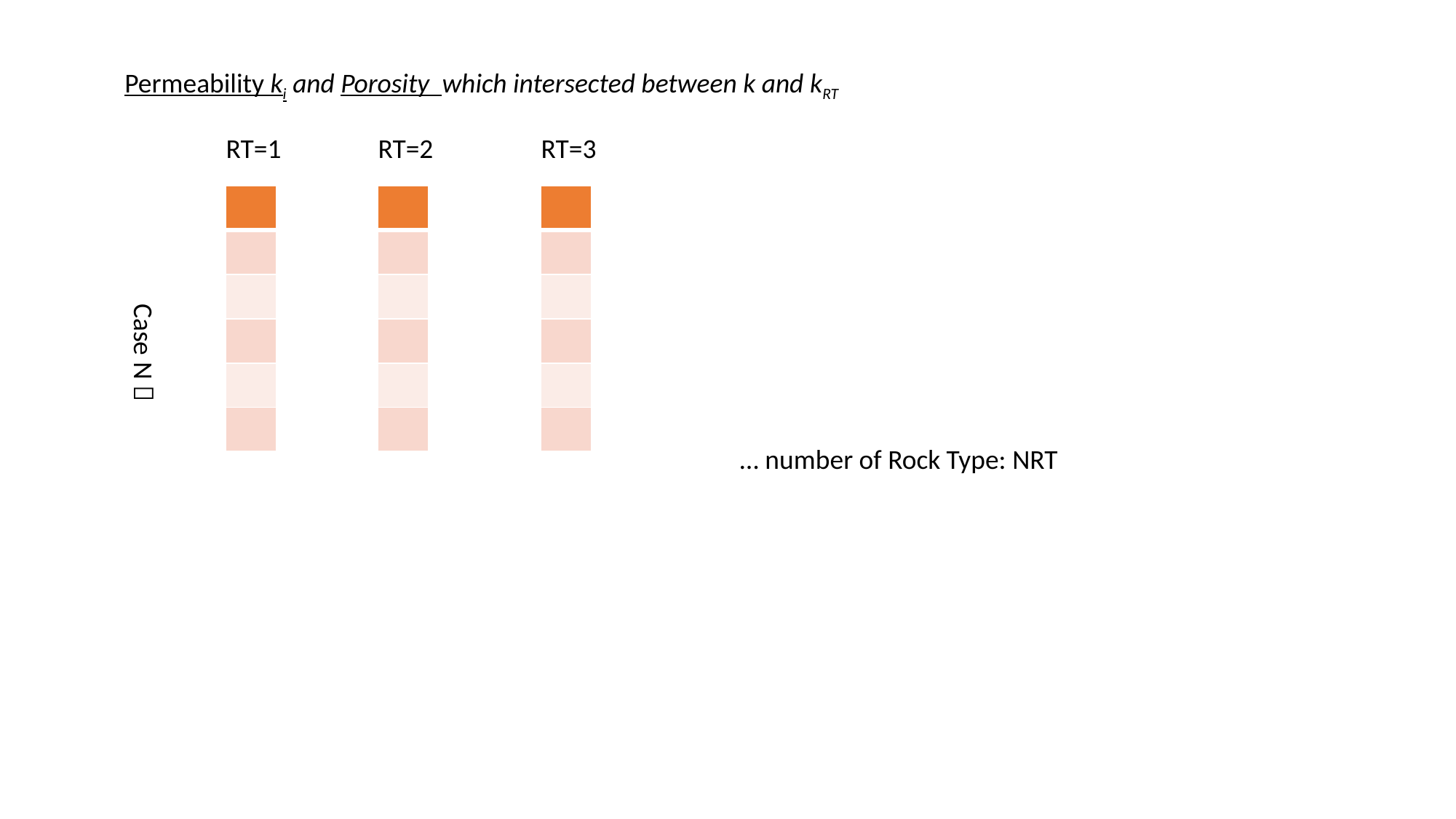

RT=1
RT=2
RT=3
| |
| --- |
| |
| |
| |
| |
| |
| |
| --- |
| |
| |
| |
| |
| |
| |
| --- |
| |
| |
| |
| |
| |
Case N 
… number of Rock Type: NRT
